# Normalizing Input–Output Relationships of Cancer Networks for Reversion Therapy

**DOI:** 10.1002/advs.202207322

**Published:** 2023-06-02

**Authors:** Jae Il Joo, Hwa‐Jeong Park, Kwang‐Hyun Cho

**Affiliations:** ^1^ Department of Bio and Brain Engineering Korea Advanced Institute of Science and Technology (KAIST) Daejeon 34141 Republic of Korea; ^2^ Present address: biorevert Inc Daejeon 34051 Republic of Korea; ^3^ Present address: Promega Corporation an affiliate of Promega South Korea

**Keywords:** Boolean networks, cancer reversion, complex networks, network control, systems biology

## Abstract

Accumulated genetic alterations in cancer cells distort cellular stimulus‐response (or input–output) relationships, resulting in uncontrolled proliferation. However, the complex molecular interaction network within a cell implicates a possibility of restoring such distorted input–output relationships by rewiring the signal flow through controlling hidden molecular switches. Here, a system framework of analyzing cellular input–output relationships in consideration of various genetic alterations and identifying possible molecular switches that can normalize the distorted relationships based on Boolean network modeling and dynamics analysis is presented. Such reversion is demonstrated by the analysis of a number of cancer molecular networks together with a focused case study on bladder cancer with in vitro experiments and patient survival data analysis. The origin of reversibility from an evolutionary point of view based on the redundancy and robustness intrinsically embedded in complex molecular regulatory networks is further discussed.

## Graphical abstract

Cancer reversion is a phenomenon where cancer cells lose their malignancy and tumorigenicity under specific conditions. Although there have been phenomenological observations on such cancer reversion, its underlying mechanism has never been investigated systematically. This study unravels the hidden principle on the reversibility of input–output relationships of complex networks that can provide a new insight into cancer reversion.

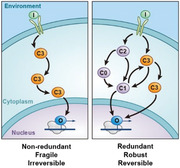

## 1 Introduction

A cell receives various stimuli from its environment and produces appropriate responses to maintain the homeostasis of the whole organism. From a system's point of view, such stimulus‐response relationships define input‐output characteristics of the cellular system that are determined by complex molecular interactions within a cell. These relationships can be distorted by genetic alterations, sometimes resulting in malignant transformation to cancer. Interestingly, the complexity of molecular interactions implicates the possibility of restoring normal input‐output relationships by compensating erroneous regulation through rewiring of the molecular interaction network. If such normalization is possible, we might be able to reprogram cancer cells to revert to normal (or normal‐like) cell states. This would open a new paradigm of reversion therapy as an alternative to current anti‐cancer therapeutics.

Cancer reversion has been sporadically observed at a phenomenological level for over a century, but the underlying mechanism is still not unveiled and no systematic method of analyzing the dynamical behavior of the underlying molecular networks has been proposed.^[^ ^1^, ^2^, ^3^, ^4^ ^]^ Therefore, this presents a renewed challenge from a systems biological perspective.^[^ ^5^ ^]^ In this study, we define the “reversion” of a cancer cell as restoring the input‐output relationships of normal cells. We investigate complex intracellular molecular regulatory networks to analyze reversibility and identify potential molecular targets to be controlled for reversion. For this purpose, we employ the Boolean network model, a logical discrete state model where a molecular activity is represented by a discretized level (high (+1) or low (0)) and the regulatory (activating or inhibiting) interaction between nodes is denoted by a link connecting the two nodes. A collection of all node activities constitutes the network state that eventually converges to a steady state called an attractor. Therefore, a subset composed of input and output nodes in an attractor represents the input‐output relationships of the cellular system at a steady state.

Even though the Boolean network model simplifies continuous biological quantities,^[^ ^6^ ^]^ it has been well proven that Boolean network models can still capture essential biological dynamics.^[^ ^7^, ^8^, ^9^, ^10^ ^]^ Because of this, there have been a number of studies on the complex network control of biological systems based on Boolean network models.^[^ ^11^, ^12^, ^13^, ^14^ ^]^ Among such studies, canalization effects between upstream and downstream nodes in Boolean networks were intensively investigated.^[^ ^14^ ^]^ Here, the canalization of a Boolean function means that its output node activity can be determined by one of multiple regulatory input nodes, called a canalizing input, irrespective of other input nodes. In this study, we employ this concept of canalization to determine the input‐output relationships of Boolean networks without the need to identify all attractor states directly, which is a very challenging problem from a computational perspective.^[^ ^15^ ^]^

On the basis of the aforementioned concepts and methods, we developed in this study a generic framework with which we can examine the reversibility of input‐output relationships of cancer cells depending on the primary driver genetic alterations. Furthermore, the framework can be used to identify molecular targets that can induce network rewiring for the restoration of distorted input‐output relationships. We examined the proposed approach with a number of cancer cell networks and validated its usefulness through both in silico and in vitro experiments with a particular focus on the example of bladder cancer cells. Patient data analysis also supports our approach. Finally, we further explored the evolutionary origin of such reversibility and found some interesting clues in terms of robustness and redundancy through extensive simulation analyses.

## 2 Results

### 2.1 Input–Output Relationships of Boolean Networks

**Figure 1:**
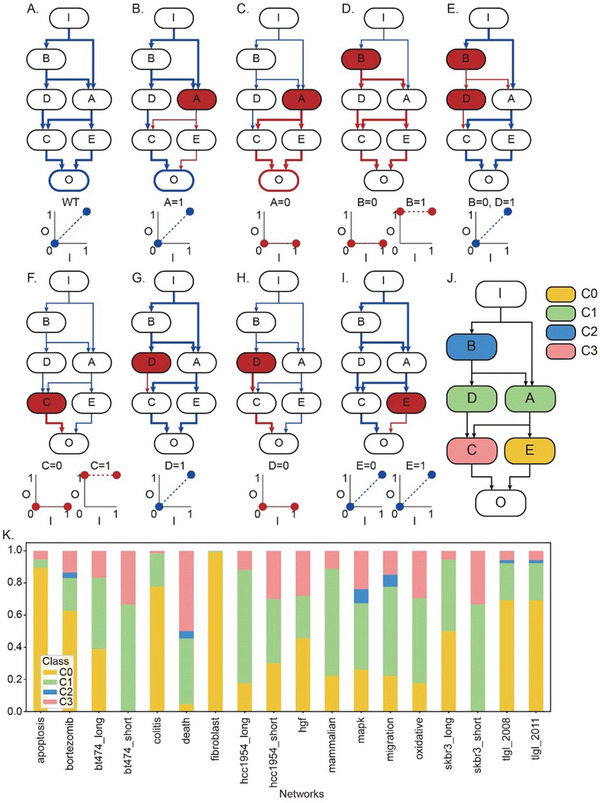
Reversibility of complex networks. A) IO relationship of an example Boolean network. The Boolean regulatory logic of each node is A(*t*+1) = I(*t*) OR B(*t*); B(*t*+1) = I(*t*); C(*t*+1) = A(*t*) AND D(*t*); D(*t*+1) = B(*t*); E(*t*+1) = A(*t*); O(*t*+1) = (E(*t*) OR C(*t*)) AND C(*t*). The color of each node represents its class according to reverse controllability. B–I) IO relationship of the mutated network. Blue links represent the signal flow from an input node, and dark red links represent the signal flow from mutated nodes in dark red color. Bold links represent dominant signal flows from the corresponding input node or mutated nodes. Blue IO relationship is normal but dark red IO relationship is disrupted. J) Node classification of the example Boolean network. The color of each node represents its class. K) Ratio of node classes of 18 Boolean networks from Cell Collective.

A cell should properly respond to extracellular stimuli to maintain homeostasis: this characteristic of a cell is defined as the input‐output (IO) relationship and is determined by the dynamics of its intracellular molecular network.^[^ ^16^ ^]^ In general, an input node of a network is defined as a node without any upstream nodes, while an output node is defined as a node without any downstream nodes. Here, we introduce Boolean network modeling to systematically analyze the dynamics of such networks and examine IO matching upon an expanded Boolean network model to determine the IO relationship with significantly reduced computational complexity (see Experimental Section for details). **Figure** **1**A illustrates the IO matching of an example Boolean network model; the state of an input node subsequently determines the states of downstream nodes following the links (their logical regulatory relationships) in blue color, ultimately determining the state of an output node. When the state of the input node is 0 (OFF), the state of the output node is determined to be 0. Likewise, when the state of the input node is 1 (ON), the state of the output node is determined to be 1. In this case, the state of the output node can be solely determined by the state of the input node, which is deterministic IO matching. However, due to the complex regulation of networks, it is not always possible to determine the states of output nodes based on the states of input nodes, resulting in nondeterministic IO matching.

If a node is fixed to a specific state by a mutation, the IO matching can be disrupted. The mutation of disrupting the IO matching is an effective mutation, otherwise, it is an ineffective mutation. In Boolean networks, each node can be mutated in two ways; the state of a node with a gain‐of‐function (GOF) mutation should be fixed to 1, whereas the state of a node with a loss‐of‐function (LOF) mutation should be fixed to 0. Interestingly, GOF mutation of the node A is ineffective whereas LOF mutation of the node A is effective (Figure 1B,C). From this case, we conceived that the disrupted IO matching by the LOF mutation on node A can be restored by fixing the state of node A to 1. Based on this, we define the “reverse control” as completely restoring the IO matching that was disrupted by a mutation to the original IO matching. If the reverse control is possible for a given mutation, then the mutation is defined as a reverse‐controllable mutation.

According to the effectiveness and reverse‐controllability of mutations, we classified the Boolean network nodes into four classes (Figure 1B–J, and Figure S2, Supporting Information). When both GOF and LOF mutations of a node are ineffective, we classified the node as a C0 node (Figure 1I). If one mutation of a node is effective whereas its complementary mutation is ineffective, then the node is classified as a C1 node (Figure 1B,C,G,H). C1 nodes are reverse‐controllable nodes since the effective mutations of C1 nodes can be reversed by controlling the nodes to be the ineffective value. Controlling a mutated node is not a special case because most targeted therapies inhibit mutated or altered genes. On the other hand, the mutation of a node can also be reversed by controlling a downstream C1 node. For instance, the LOF mutation of node B can be reversed by controlling node D to be 1 (Figure 1E). The GOF mutation of node B is also reverse‐controllable because the original IO matching can be restored by controlling node B and D to be 0 and 1, respectively. If these nodes are not C1 nodes, then we classify them as C2 nodes. Finally, the remaining nodes are classified as C3 nodes, which are not reverse‐controllable (Figure 1F).

### 2.2 Reversibility of Cancer Boolean Networks

**Figure 2:**
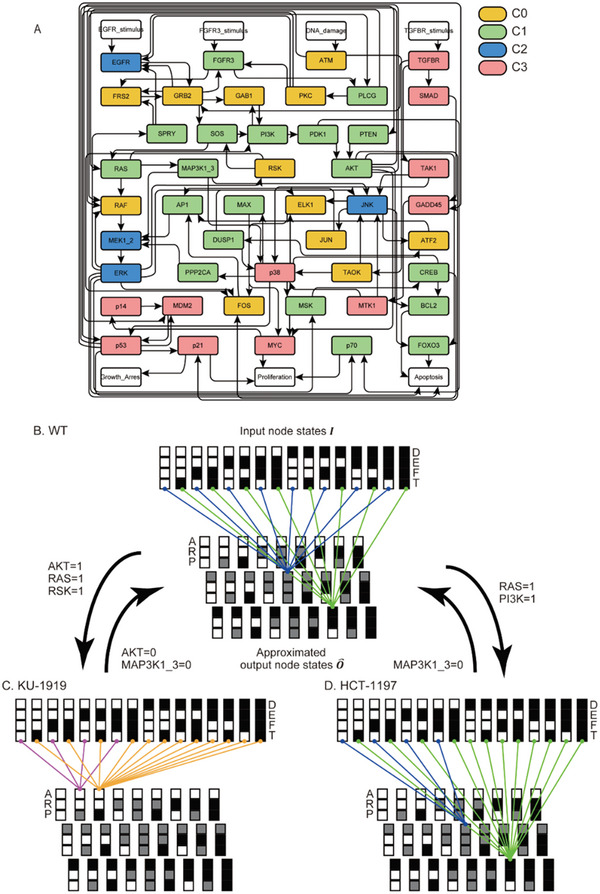
Reversibility of a bladder cancer network. A) A bladder cancer Boolean network (“mapk” network). The color of each node represents its class. IO relationship of B) wild type (WT), C) the KU‐1919 cancer cell line and D) the HCT‐1197 cancer cell line. Each stacked square represents an input node state and an inferred output node state (white: 0, gray: 0.5, black: 1). The lines between input node states and inferred output node states represent the IO matching (D: DNA_damage, E: EGFR_stimulus, F: FGFR3_stimulus, T: TGFBR_stimulus, A: Apoptosis, R: Grwoth_Arrest, P: Proliferation). Lines with the same color are connected to the same inferred output node state from various input node states. The IO matching of the KU‐1919 and HCT‐1197 cell lines are obtained by applying mutations [AKT = 1, RAS = 1, RSK = 1] and [RAS = 1, PI3K = 1], respectively. The disrupted IO matching of KU‐1919 and HCT‐1197 can be normalized to that of WT by controlling node states [AKT = 0, MAP3K1_3 = 0] and [MAP3K1_3 = 0], respectively.

By applying the node classifications based on reverse‐controllability, we analyzed 18 Boolean networks from the Cell Collective which are related to cancer or cell fate decision‐making (Table S1 and Figure S1, Supporting Information).^[^ ^17^, ^18^, ^19^, ^20^, ^21^, ^22^, ^23^, ^24^, ^25^, ^26^, ^27^, ^28^, ^29^, ^30^ ^]^ We found that most networks have less than 40% C3 nodes, which indicate the reversibility of cancer networks (Figure 1K). To examine the reversibility of cancer networks in more details, we further analyzed the bladder cancer Boolean model (“mapk” network)^[^ ^25^ ^]^ since the bladder cancer model shows strong dependency on the TGF‐b signal, and thus in vitro experiments for the validation of IO matching restoration are possible (**Figure** **2**A,B). The bladder cancer network contains four input nodes, three output nodes, and 46 internal nodes. According to reverse‐controllability, the 46 internal nodes are classified into 12 C0 nodes, 19 C1 nodes, 4 C2 nodes, and 11 C3 nodes (Figure 2A). From the OncoKB database, we investigated the cancer genes in the bladder cancer network (**Table** **1**).^[^ ^31^ ^]^ Among the 12 C0 nodes, only 4 nodes are known as cancer genes. However, in the case of the other classes, more than half of the nodes are cancer genes: 13/19 for C1, 3/4 for C2, and 7/11 for C3. As mutations on C0 nodes are ineffective, we can infer that C0 nodes might be less related to cancer genes than other node classes.

**Table 1:** Class, gene name, and annotation by OncoKB of each node in the bladder cancer network

Node	Class	Gene name	OncoKB
ATF2	C0	ATF2	
ATM	C0	ATM	Tumor suppressor
ELK1	C0	ELK1	
FOS	C0	FOS	
FRS2	C0	FRS2	
GAB1	C0	GAB1	Oncogene
GRB2	C0	GRB2	
JUN	C0	JUN	Oncogene
PKC	C0	PRKCA PRKCB PRKCG	
RAF	C0	RAF1 BRAF ARAF	Oncogene
RSK	C0	RPS6KA1 RPS6KA2 RPS6KA3 RPS6KA6	
TAOK	C0	TAOK1 TAOK2 TAOK3	
AKT	C1	AKT1 AKT2 AKT3	Oncogene
AP1	C1		
BCL2	C1	BCL2	Oncogene
CREB	C1	CREB1	Oncogene
DUSP1	C1	DUSP1	
FGFR3	C1	FGFR3	Oncogene
FOXO3	C1	FOXO3	
MAP3K1_3	C1	MAP3K1 MAP3K2 MAP3K3	Tumor suppressor
MAX	C1	MAX	Tumor suppressor
MSK	C1	RPS6KA4 RPS6KA5	Oncogene
p70	C1	RPS6KB1 RPS6KB2	Oncogene
PDK1	C1	PDK1	
PI3K	C1	PIK3CA	Oncogene
PLCG	C1	PLCG1 PLCG2	Oncogene
PPP2CA	C1	PPP2CA	
PTEN	C1	PTEN	Tumor suppressor
RAS	C1	HRAS KRAS NRAS	Oncogene
SOS	C1	SOS1 SOS2	Oncogene
SPRY	C1	SPRY2	
EGFR	C2	EGFR	Oncogene
ERK	C2	MAPK1 MAPK3	Oncogene
JNK	C2	MAPK8 MAPK9	
MEK1_2	C2	MAP2K1 MAP2K2	Oncogene
GADD45	C3	GADD45A GADD45B GADD45G	
MDM2	C3	MDM2	Oncogene
MTK1	C3	MAP3K4	
MYC	C3	MYC	Oncogene
p14	C3	CDKN2A	Tumor Suppressor
p21	C3	CDKN1A	Tumor Suppressor
p38	C3	MAPK11 MAPK12 MAPK13 MAPK14	
p53	C3	TP53	Tumor Suppressor
SMAD	C3	SMAD2 SMAD3 SMAD4	Tumor Suppressor
TAK1	C3	MAP3K7	
TGFBR	C3	TGFBR1 TGFBR2 TGFBR3	Tumor Suppressor

We examined the efficacy of reverse control targets of bladder cancer cell lines. Most bladder cancer cell lines have genetic alterations on C3 nodes, whereas KU‐1919 and HT‐1197 cell lines have genetic alterations on reversible nodes only (**Table** **2**). We rewired the bladder cancer network by mapping genetic alterations of the corresponding cell lines and then evaluated the effects of C1 node interventions within the rewired networks. As a result, we found that simultaneous inhibition of both MAP3K1 and AKT can restore the disrupted IO matching of KU‐1919 cells and that inhibition of MAP3K1 is sufficient to restore the disrupted IO matching of HT‐1197 cells (Figure 2C,D).

**Table 2:** Genetic alteration profile of bladder cancer cell lines. Each row represents a genetic profile of a bladder cancer line. Columns are names of genes. GOF: gain‐of‐function, LOF: loss‐of‐function

	AKT1	ATM	CDKN1A	CDKN2A	FGFR3	HRAS	KRAS	MAPK1	MAPK3	MAX	MYC	NRAS	PIK3CA	PTEN	RPS6KB2	TP53
BC3C													GOF			LOF
BFTC905											GOF	GOF				LOF
CAL29													GOF		GOF	LOF
HT1197												GOF	GOF			
HT1376			LOF						GOF							LOF
J82					GOF								GOF			LOF
JMSU1		LOF														LOF
KMBC2			LOF							LOF				LOF		LOF
KU1919	GOF											GOF			GOF	
RT112			LOF		GOF								GOF			LOF
RT11284																LOF
SCABER				LOF												LOF
SW1710																LOF
T24						GOF										LOF
TCCSUP													GOF			LOF
UMUC1																LOF
UMUC3		LOF					GOF									LOF
VMCUB1				LOF				GOF					GOF			LOF

**Figure 3:**
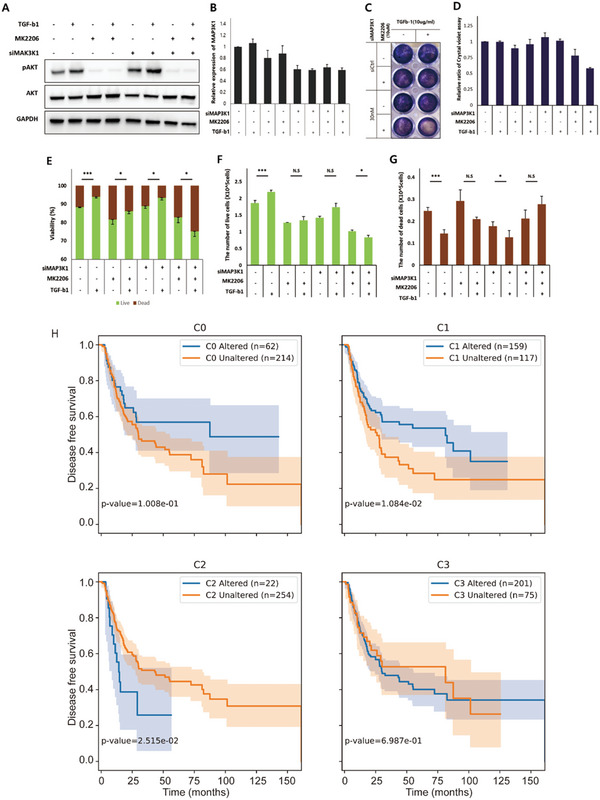
Biological supporting evidence for the reversibility of the bladder cancer network. siMAP3K1‐transfected cells and untransfected KU‐1919 cells were grown with MK2206 and TGF‐b for 48 h. A,B) KU‐1919 cells of each group were subjected to western blot assays and RT‐qPCR. C,D) KU‐1919 cells were stained for crystal violet assays and the intensity of the staining was measured. E,F), and G) KU‐1919 cell viability was determined by trypan blue dye. Statistically significant differences were determined by a two‐tailed Student's t‐test (*n* = 4): *p < 0.05 and ***p < 0.005. Error bar represents standard errors. H) Disease‐free survival analysis of TCGA bladder cancer patients (*n* = 276) according to alterations in each node class.

Compared to the normal network, the KU‐1919 network responds differently to TGF‐b (Figure 2B,C). TGF‐b is well known to exert anti‐proliferative effects and regulate differentiation during normal development in various organs in vertebrates.^[^ ^32^ ^]^ In contrast, growth inhibition via TGF‐b signaling is released and TGF‐b instead promotes invasion and metastasis in some cancers.^[^ ^33^ ^]^ Likewise, KU‐1919 showed evading growth inhibition via TGF‐b signal in both our simulation of the Boolean network model and experimental validation (**Figure** **3**A–G). To validate the IO relationship restoration of the KU‐1919 cells, we investigated the influences of MAP3K1 and AKT inhibition on the regulation of cellular response to TGF‐b in KU‐1919 cells by using siRNAs against MAP3K1 and MK2206 (a specific inhibitor of AKT). Combined treatment of MAP3K1 siRNAs and MK2206 further reduced the intensity of crystal violet staining of KU‐1919 cells attached to the culture plates when TGF‐b was included in the culture media; this indicates that the simultaneous blockage of both components induces a normal response to TGF‐b stimulation. To further determine whether the co‐inhibition of MAP3K1 and AKT induces an increase of cell death, dead cells were counted separately via the trypan blue assay. The results indicated that, unlike the responses in the control and/or single inhibition groups, the decreased activity of both of MAP3K1 and AKT contributed to increasing the number of dead cells, ultimately lowering viability through TGF‐b stimulation.

We further examined the restoration of IO relationship using other human bladder carcinoma cell line, HT‐1197. Compared to the normal network, the HT‐1197 network exhibits sensitivity to DNA damage (Figure 2B,D). siRNA‐mediated depletion of MAP3K1 caused a reduction in cytotoxicity induced by fluorouracil in the HT‐1197 in vitro experiment (Figure S3, Supporting Information). Moreover, less nuclear foci consisting of phosphorylated histone H2AX—a sensitive marker of DNA damage response (DDR)—were observed in MAP3K1 siRNA‐treated cells than in untreated cells.^[^ ^34^ ^]^ These results indicate that inhibition of MAP3K1 expression may interfere with proper DDR, which stops DNA replication and prevents the transmission of damaged genetic information to daughter cells. From these results, we concluded that IO matching restoration via reverse control can be reliably achieved in cancer cells as expected from the simulation analysis.

We also performed the Kaplan‐Meier analysis of the disease‐free survival rate of bladder cancer patients. Patients (*n* = 276) were grouped into altered or unaltered groups according to whether they have alterations on each node class or not. Intriguingly, we found that patients with alterations of C1 nodes have better disease‐free survival rates than patients without C1 node alterations (altered = 159, unaltered = 117, *p*‐val = 0.01) (Figure 3H, top‐right). On the other hand, patients with C2 node alterations have worse disease‐free survival rates than patients without C2 node alterations (altered = 22, unaltered = 254, *p*‐val = 0.02) (Figure 3H bottom‐left). Lastly, C0 and C3 node alterations have insignificant effects on patient survival (Figure 3H, top‐left and bottom‐right). In terms of overall survival, alterations of each node class show insignificance among the altered and unaltered groups (Figure S4, Supporting Information).

The bladder cancer patient analysis results indicate the possibility that alterations of the C1 node class might be beneficial to patient survival. To further investigate this, we analyzed other cancer networks. In the breast cancer network (“mammalian” network), there are 4 C0 nodes, 12 C1 nodes, and 2 C3 nodes. We analyzed overall, progression‐free, and disease‐free survival of breast cancer patients and found that C1 alterations (altered = 192, unaltered = 137) have beneficial effects on overall (*p*‐val = 0.02) and progression‐free survival (*p*‐val = 0.01) but an insignificant effect on disease‐free survival (Figure S5, Supporting Information). To further validate our method, we applied the method to another cancer network model. Recently, Choi et al. found that inhibition of BCL11A and HDAC1/2 can reprogram basal‐like breast cancer cells into luminal A breast cancer cells, which induces sensitivity to endocrine therapy.^[^ ^8^ ^]^ They constructed a Boolean network model that can represent basal and luminal A breast cancer cells with two input nodes, EGF and Estrogen. By applying our method, the nodes in their Boolean network are classified as follows: ER, CCNE1, FOXC1, HDAC1/2 and BCL11A are classified as C1 nodes; the rest nodes are classified as C0 nodes (Figure S6, Supporting Information). Choi et al. showed in their experiments that inhibition of BCL11A or HDAC1/2 results in partial normalization in response to tamoxifen (an estrogen receptor inhibitor). They also showed that inhibition of BCL11A and HDAC1/2 can synergistically induce responsiveness to tamoxifen. These support the prediction of our method. Moreover, they showed that low expressions of BCL11A and HDAC1/2 are correlated with better prognosis of breast cancer patient, which supports the clinical benefits of IO relationship normalization.

### 2.3 Robustness, Redundancy, and Reversibility of Boolean Networks

Many nodes of Boolean networks in the Cell Collective database are reverse‐controllable. Based on this, we hypothesized that intracellular molecular interaction networks might have gained more reverse‐controllable nodes and thereby possess high reversibility during the evolutionary process. As it is known that such intracellular networks acquired robustness and redundancy through evolution,^[^ ^35^, ^36^, ^37^, ^38^, ^39^, ^40^ ^]^ we tried to identify the relationships among the reversibility, redundancy, and robustness of complex molecular interaction networks. Theoretically, any complex network without redundancy in IO paths (such as a simple cascade) should be irreversible and therefore fragile to random mutation.

For quantitative analysis, we defined measures for the reversibility, redundancy, and robustness of a Boolean network (see Experimental Section for detailed information). First, the reversibility of a network is measured by the ratio of C0, C1, and C2, excluding C3 nodes. If a network has a mutation on a C3 node, the IO matching of the network cannot be restored. In other words, a network is less reversible if it has more C3 nodes than other networks. Secondly, the redundancy of a network is measured by the number of paths between input and output nodes that have deterministic IO matching. Finally, we defined two measures for the robustness of a network. The robustness to mutations is measured by the average of the ratios of retained primary attractors after one node mutation, whereas the robustness to perturbation is measured by the ratio of initial states converging to the same attractor independent of a single node perturbation.

**Figure 4:**
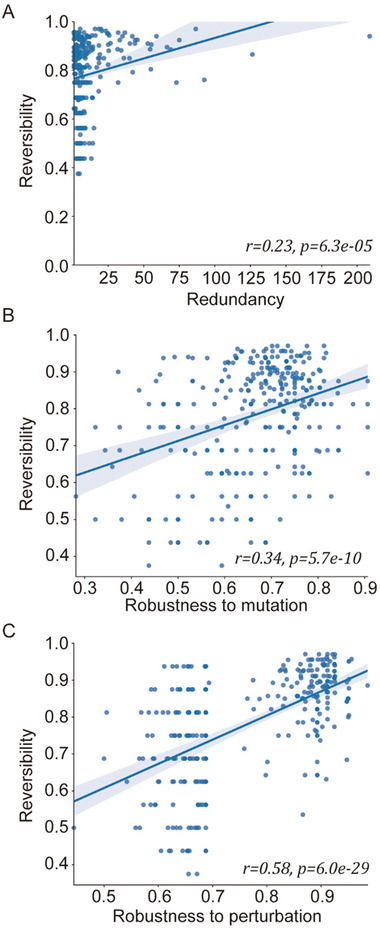
Relationships among reversibility, robustness, and redundancy of random complex networks. A) Reversibility and redundancy. B) Reversibility and robustness to a permanent mutation. C) Reversibility and robustness to a transient perturbation. Random complex networks are generated by rewiring 10 Cell Collective Boolean networks with conserved nodal degree distribution. Pearson correlation *r* and *p*‐value are represented on each graph. The shaded area represents the 95% confidence interval for linear regression (*n* = 306).

We analyzed the robustness, redundancy, and reversibility measures of the Cell Collective networks and found that they have positive tendencies but are not much significant (Figure S7, Supporting Information). In this analysis, five networks were excluded as they have no deterministic IO matching. To validate the significance of such tendency, we generated 100 random configuration models for each of the 10 Cell Collective networks since the rest of networks take too much time to generate random models (Table S1, Supporting Information). Then, we measured the robustness, redundancy, and reversibility of 306 networks among 1000 networks which have at least one deterministic IO matching (Table S2, Supporting Information). As we expected, the redundancy and reversibility of the networks exhibited a positive correlation (*r* = 0.23, *p*‐val = 6.3 × 10^−5^, Pearson; *r* = 0.21, *p*‐val = 0.00026, Spearman; **Figure** **4**A). Furthermore, reversibility has positive correlations with robustness to mutations (*r* = 0.34, *p*‐val = 5.7 × 10^−10^, Pearson; *r* = 0.28, *p*‐val = 1.0 × 10^−6^, Spearman; Figure 4B) as well as robustness to perturbation (*r* = 0.58, *p*‐val = 6.0 × 10^−29^, Pearson; *r* = 0.57, *p*‐val = 6.2 × 10^−28^, Spearman; Figure 4C).

## 3 Discussion

Cancer has been considered irreversible since genetic alterations are irreversible, and thereby some attempts to artificially restoring altered gene activity have been suggested for cancer reversion.^[^ ^4^ ^]^ However, there is a growing amount of evidence supporting the idea that cancer reversion might be possible without directly restoring mutated gene activities. For instance, it was recently revealed that a normal cell can maintain its normal identity even after it acquires oncogenic driver mutations.^[^ ^41^, ^42^ ^]^ Moreover, some molecular targets that can induce cancer reversion without gene restoration were identified and experimentally validated.^[^ ^3^, ^43^, ^44^, ^45^ ^]^ Nonetheless, no systematic framework has been available to date with which researchers can investigate cancer reversion at a molecular regulation level. In this study, we expounded that the IO relationships of genetically perturbed intracellular molecular networks can be normalized through elaborate control under certain specific conditions and that the reversibility of a generic complex network is related to the intrinsic robustness and redundancy of the network. Considering the similar properties across various intracellular molecular networks, we expect that the distorted IO relationships of many other intracellular molecular networks might also be reversible through some proper control of perturbed network dynamics.

Our method is the first approach to examine the IO relationship and to control the disrupted dynamics of a damaged network caused by genetic alterations. Other previous control methods cannot be used for analyzing IO relationship because most of these methods focus on how to drive a network state to a single desired state which cannot represent the IO relationship. Nevertheless, we compared our method with the most relevant study that classified the nodes of a complex network into indispensable, dispensable, and neutral nodes.^[^ ^46^ ^]^ Vinayagam et al. classified a node as indispensable if its removal increases the number of driver nodes required to fully control the complex network. Conversely, they classified a node as dispensable if its removal decreases the number of driver nodes. If removal of a node has no effect, it was classified as neutral. Driver nodes include all the input nodes and thus their change can be considered as an interruption of IO relationship. By using this method, we analyzed and classified the nodes of 18 networks from the Cell Collective, which are shown in Figure 1K and Figure S1, Supporting Information and compared with the results of our study. It shows that there is no correlation between them (Figure S8, Supporting Information), since Vinayagam et al. only considered the change of the number of driver nodes when a node is removed, not the IO relationship.

Recently, intracellular molecular regulatory network models, including Boolean network models, are becoming more available with the rapid development of high‐throughput measurement technologies such as single cell‐resolution transcriptomic measurements and various network reconstruction methods based on these data.^[^ ^47^, ^48^, ^49^, ^50^ ^]^ When a Boolean network model of intracellular molecular regulations is given, our method automatically classifies the given network nodes and identifies therapeutic reversion targets without any prior knowledge. However, regulatory logic information of a network model may contain more uncertainty compared to interaction structures. Thus, we have further investigated whether our method can still provide consistent results under such uncertainty. For this purpose, we have analyzed the bladder cancer network by introducing various degrees of uncertainty in the regulatory logics and confirmed that our method can consistently (>80%) classify the network nodes with about 5% of random alteration of regulatory logics in the network model (Figure S9, Supporting Information).

From an evolutionary point of view, it seems that there is no reason for intracellular networks to acquire such reversibility through evolution since the restoration of IO relationships needs an exquisite event that can counteract mutational effects, which would be very unlikely to occur under natural circumstances. On the other hand, some previous studies showed that biological networks might have acquired redundancy while achieving robustness of their regulatory functions under noisy environments during evolution.^[^ ^35^, ^36^, ^37^, ^38^ ^]^ As our results on complex network analysis further show correlations between reversibility and both redundancy and robustness, we infer that the evolutionary origin of reversibility might be the intrinsic redundancy of intracellular molecular networks achieved as a byproduct during network evolution towards robustness for homeostasis.

Although a single mutation is usually considered to classify the nodes of Boolean networks, actual cancer cells have numerous genetic mutations and alterations. To apply our method to such cases, it is necessary to infer functional mutations and alterations of a cancer patient to assess whether the patient has any alteration on C3 genes. If the patient does not have a C3 gene alteration, then we can identify the reverse control targets by analyzing the combined effect of reversion targets based on the information of genetic alterations.

It is still possible that a genetic profile of a patient is irreversible without mutations on C3 genes. For instance, the breast and bladder cancer networks are reversible for about 90% of combinations of three mutations on reversible nodes but not for the rest 10% combinations^[^ ^24^, ^25^ ^]^ (Figure S10, Supporting Information); three mutations in this analysis correspond to about 20% and 10% damage on the breast and bladder cancer networks, respectively. Cancer patients may have less than 10% mutated and altered genes in many cases.^[^ ^51^ ^]^ Therefore, our reverse control strategy for cancer reversion might be sufficiently reliable. In future studies, we will need to further investigate the maximum number of mutations that can allow for reversibility at the whole‐genome level.

We classified the nodes of various molecular regulatory networks into 4 classes. In particular, C1 nodes are classified as promising targets for cancer reversion. By regulating C1 nodes, the mutational effect of upstream C1 or C2 nodes can be compensated. Interestingly, bladder cancer patients carrying alterations or mutations on C1 genes showed better survival scores than others. On the other hand, alterations or mutations on C2 genes showed the opposite result to that of C1 genes. C1 and C2 genes are both reversible by regulating downstream C1 genes, whereas C1 genes are also reversible by regulating themselves. Since most targeted therapies share targets and biomarkers, targeted therapy for C1 altered groups might have had some similar favorable effects to those expected of cancer reversion, thereby resulting in such promising outcomes. On the other hand, targeted therapy for C2 altered groups might have been successful in inducing apoptosis or arrest of cancer cells, but since it cannot restore IO matching, the targeted therapy might have incurred significant adverse effects on normal cells. C3 genes are the biomarker for irreversible genetic profiles. If a cancer patient carries mutations of C3 genes, the complete restoration of IO matching would not be possible. In this case, we can consider partial restoration of IO matching. Since some stimuli induce cell cycle arrest or apoptosis, restoration of the dependency of these stimuli is the focus for the partial restoration of IO matching and has been under study to sensitize cancer cells to anti‐cancer drugs.^[^ ^52^, ^53^ ^]^ For instance, if we restore dependency to a growth stimulus, then cancer cells can be treated with inhibitors of growth stimulus responses.^[^ ^8^ ^]^

We identified reversion targets, AKT and MAP3K1_3 in bladder cancer cell lines. Here, AKT has been suggested as a therapeutic target for cancer,^[^ ^54^ ^]^ and there has been success in clinical trial by combinatorial therapy using a chemotherapeutic agent and an AKT inhibitor in metastatic breast cancer.^[^ ^55^ ^]^ This suggests possible clinical applications of this target in other cancer types, including bladder cancer. On the other hand, MAP3K family genes have been suggested cancer‐association factors,^[^ ^56^, ^57^, ^58^ ^]^ but there is no clinical trial directly related to this target due to lack of selective MAP3K inhibitors. In addition, there are other known reversion therapeutic targets, TPT1/TCTP and SETDB1 from previous studies.^[^ ^2^, ^3^, ^43^ ^]^ Although these studies do not provide Boolean network models, we can still suggest that the cancer reversion induced by inhibiting each target is comparable with the normalization of IO relationship. TPT1/TCTP is the first target for inducing cancer reversion, which is currently in a clinical trial.^[^ ^59^ ^]^ This gene forms a feedback loop with P53 which is known to mediate density‐dependent growth arrest.^[^ ^60^ ^]^ In this case, we can presume that the density is an input node and the growth arrest is an output node of a subnetwork within the entire complex P53 network. When TPT1/TCTP is highly expressed, P53 cannot mediate the density‐dependent growth arrest, which can be considered as an interruption of IO relationship. When TPT1/TCTP is repressed, however, P53 can mediate the density‐dependent growth arrest, which can be interpreted as the normalization of IO relationship.^[^ ^60^ ^]^ Hence, we can classify TPT1/TCTP as a C1 node. Finally, Lee et al. found that expression levels of the identified five transcription factors were similar in normal and cancer cells even though these cells had differentially expressed genes that are regulated by these transcription factors.^[^ ^43^ ^]^ SETDB1 was identified as the potential mediator of these transcription factors in cancer cells. In this case, the five transcription factors can be considered as input nodes and the genes that are differentially expressed in normal and cancer cells can be considered as output nodes of the underlying gene regulatory network. Here, SETDB1 can be classified as a C1 node since its expression interferes with the IO relationship whereas its inhibition normalizes the IO relationship. Low expressions of TPT1/TCTP and SETDB1 are correlated in leading to a better prognosis of breast cancer and colon cancer patients, respectively, which supports clinical benefits of the IO relationship normalization.^[^ ^43^, ^60^ ^]^

Cancer reversion might be a promising future cancer treatment strategy that can be an alternative to current cancer therapy which aims at only killing cancer cells. Current anti‐cancer therapies have inevitable side effects such as the death of normal stem cells. In contrast, cancer reversion would have much less (or negligible) side effects as it does not aim at killing cancer cells. Although cancer immunotherapy and targeted therapy have fewer side effects than traditional cytotoxic drugs, the aim of these treatment strategies is still to kill cancer cells, and thus the applicability of these methods is limited. In contrast, the targets of cancer reversion are C1 genes, and interventions on these genes have no effect on the IO matching of normal cells. On the other hand, interventions on C2 or C3 genes would interfere with normal IO matching and thereby cause side effects by inducing cell cycle arrest or apoptosis of normal cells. It is also worthwhile to note that identified targets for cancer reversion in many cases would be novel drug targets. As cancer reversion does not directly induce the death of cancer cells, targets for cancer reversion would have been neglected during the target identification process of conventional anti‐cancer drug discovery. By changing the concept of cancer therapy, we can identify novel drug targets and have new opportunities to develop completely different therapies for cancer patients. We also need to note that any anti‐cancer drugs that induce apoptosis would accelerate the clonal evolution of cancer cells and eventually result in acquired resistance. In contrast, the cancer reversion approach would maintain cancer cell populations and suppress their growth, affecting only the fitness of natural selection. Hence, cancer reversion would not incur such acquired resistance.

## 4 Experimental Section

### Boolean Network Models and Expanded Networks

The Boolean network model, which is the historically oldest discrete state logical models for dynamical systems, was proposed by Kauffman.^[^ ^61^ ^]^ Boolean networks represent the dynamics of an influence (regulatory) network in which nodes influence the activity values of each other via edges. Nodes can take the state being off or on, taking the Boolean values 0 or 1, respectively; edges represent the regulatory interactions (influences) between two nodes and represent the modality of the influence (e.g., activation or inhibition). The latter is formalized by the Boolean function *f_i_* associated with node *i*, which integrates the values of its input nodes to update its value. Thus, to represent the states of a Boolean network, a state vector ***x*** (*t*) at specific time *t* is defined as ***x*** (*t*) = (*x*_1_(*t*), …*x_i_* (*t*),…, *x_N_* (*t*)); *N* is the number of nodes of a network and *x_i_* (*t*) is a state of the *i*‐th node at time *t*. Because the value of a node is either 0 or 1, ***x*** (*t*) is represented as an *n*‐bit binary vector. The value of the *i*‐th node is updated at each discrete time step by a Boolean function $f_{i}\left(x_{j\in I_{i}}\right)$; *I_i_* is the set of upstream regulators that serve as inputs of the *i*‐th node and $x_{j\in I_{i}}$ is a state vector of all regulating nodes. Thus, $x\;\left(t+1\right)=\;\left(f_{1}\left(x{\left(t\right)}_{j\in I_{1}}\right),\;\text{…}f_{i}\left(x{\left(t\right)}_{j\in I_{i}}\right),\text{…},\;f_{N}\left(x{\left(t\right)}_{j\in I_{N}}\right)\right)$. If ***x*** (*t* + *L*) = ***x*** (*t*), then the sequence of states from ***x*** (*t*) to ***x*** (*t* + *L* − 1) is a cyclic attractor for *L* > 1. If *L* = 1 and thus ***x*** (*t* + 1) = ***x*** (*t*), then ***x*** (*t*) is a point attractor. When the *i*‐th node is mutated, the Boolean function of the mutated node is $f_{i}^{{}^{\prime }}\;\left(x_{j\in I_{i}}\right)=\;0$ or $f_{i}^{\prime }\;\left(x_{j\in I_{i}}\right)=\;1$ for an LOF mutation or GOF mutation, respectively.

An expanded network is a graphical representation of the logical dynamics of a Boolean network.^[^ ^14^ ^]^ Nodes of the expanded network represent the states of nodes of the original Boolean network, hence the expanded network has at least a double number of nodes compared to the original network. Each link of the expanded network represents the sufficient relationship between node states: simply OR logic. If a node of the expanded network has only one incoming link, then the start node of that link is the sufficient and necessary condition. The expanded network has composite nodes that work as AND logic gates, and thus the start node of an incoming link to a composite node is a necessary condition for the end nodes of an outgoing link from the composite node. The LDOI of an intervention can be obtained by iteratively searching nodes downstream of the intervened node on an expanded network using a modified breadth‐first search (BFS)—which is different from the original BFS—to handle the composite nodes.^[^ ^14^ ^]^

### Input–Output Matching of Boolean Networks

The IO relationship of a Boolean network can be represented by a function *R*: *I* = {0, 1}^*l*^ ↦ ***O*** = [0, 1]^m^ , with *l* ≥ 1 input node states $I\;={\left\{I_{i}\right\}}_{i=1}^{l}\;$ and *m* ≥ 1 output node states $O\;={\left\{O_{j}\right\}}_{j=1}^{m}$. To figure out the IO relationship of a Boolean network, the states of input and output nodes are needed to identify from every attractor state. However, identifying all attractor states of a Boolean network is known as an NP‐hard problem because the number of all possible initial states is 2*^N^* for a Boolean network with *N* nodes. To overcome such complexity, the logical domain of influence (LDOI) was applied, which is a sufficient set of stabilized node states, by fixing states of certain nodes since LDOI can represent canalizing effects of Boolean functions without regard to the initial state of nodes.^[^ ^14^ ^]^ By using LDOI, the IO relationship of a Boolean network can be inferred and defined it as “IO matching” such that $M:I\mapsto \;\hat{O}={\left\{0,\;0.5,\;1\right\}}^{m}$ with inferred states of output nodes $\hat{O}={\left\{{\hat{O}}_{j}\right\}}_{j=1}^{m}$.

The IO matching $M:I\mapsto \hat{O}$ is defined by employing the LDOI as follows:

(1) $${\hat{O}}_{j}=\left\{\begin{array}{c} 0,\;if\;\left\{O_{j}=0\right\}\in LDOI\left(I\right)\\ 1,\;if\;\left\{O_{j}=1\right\}\in LDOI\left(I\right)\\ 0.5,\;if\;\left\{O_{j}=0\right\}\notin LDOI\left(I\right)and\;\left\{O_{j}=1\right\}\notin LDOI\left(I\right) \end{array}\right.\;$$

As LDOI is a sufficient set of the stabilized node states (*10*), IO matching is sufficient for IO relationships. ${\hat{O}}_{j}=\;1$ (or 0) implies *O_j_* = 1 (or 0), and if ${\hat{O}}_{j}=\;0.5$, then it implies that 0 < *O_j_* < 1. When an input node state is matched to the inferred states of all output nodes as 0.5, it is defined as nondeterministic IO matching. Otherwise, it is defined as deterministic IO matching.

The complexity of LDOI is bounded by *O*(*k* ^2^ *N*)^[^ ^14^ ^]^ where *k* is the average of in‐degree distribution and *N* is the number of nodes in a network. Our method considers all possible input node states, and thereby approximation of IO relationship is bounded by *O*(2^*I*^ × *k* ^2^ *N*) where *I* is the number of input nodes in the network. Finally, the method is bounded by *O*(2*N* × 2^*I*^ × *k* ^2^ *N*) for classifying C0 and C1 nodes, which requires simulation for all possible single node mutation *2N*, and bounded by *O*(4*N* ^2^ × 2^*I*^ × *k* ^2^ *N*) for classifying C2 nodes which require simulations for double mutations. C3 nodes are the rest of the nodes except C0, C1 and C2 nodes, and thus no additional computation is required. Hence, the node classification of our method is bounded by *O*(2^*I*^ *k* ^2^ *N* ^3^), and our method can be applied in analyzing a large‐scale Boolean network model since the complexity can be estimated by a polynomial of *N* and *k* where *I* is mostly a small number.

### Random Configuration Boolean model

To analyze robustness, redundancy, and reversibility, 1000 random directed configuration networks were generated from the degree distribution of 10 Cell Collective networks (100 random networks for each Cell Collective network, Tables S1 and S2, Supporting Information).^[^ ^62^ ^]^ Boolean functions for random networks are defined as canalizing Boolean functions as follows:

(2) $$f_{i}\;\left(x_{j\in I_{i}}\right)=\left\{\begin{array}{c} a,\;if\;x_{c}=b\;and\;c\in I_{i}\\ g\;\left({\left\{x_{j}\right\}}_{j\neq c}^{I_{i}}\right) \end{array}\right.\;$$

with the canalizing variable *x_c_*, the canalized value *a*, and the canalizing value *b*, which are determined randomly. *g* is a random Boolean function with bias = 0.5 (e.g., unbiased random Boolean function).^[^ ^63^ ^]^ Random alterations in the bladder cancer Boolean network model are based on Equation (2).

### Robustness, Redundancy, and Reversibility of a Boolean network

The reversibility of a network is correlated to the ratio of C0, C1, and C2 nodes. Thus, the reversibility *V* of a network is defined as:

(3) $$V\;=\;1-\;\frac{N_{T}}{N}$$

*N_T_* is the number of C_3_ nodes in the network and *N* is the number of nodes in the network. The number of simple paths between input and output nodes determines the redundancy *D* of a network. However, some pairs of input and output nodes are not matched as the states of these output nodes are dependent on the states of the internal nodes. Thus, simple paths were only dealt from every input node *i* to matched output nodes *j* (${\hat{O}}_{j}\neq 0.5$):

(4) $$D\;=\frac{N_{\mathrm{sp}}}{N_{p}}\;$$

*N*_sp_ is the number of simple paths from every input node to matched output nodes, and N_p_ is the number of pairs of input nodes and matched output nodes. As IO matching is dependent on the states of the input nodes, an input node can be matched to an output node twice. Considering the excessive computation time to be taken to identify all simple paths, simple paths were identified that are shorter than twice the length of the shortest path between each input and output node.

The robustness to a mutation is measured by the average of the ratio of retained primary attractors after one node mutation. 2^10^ initial states were sampled to identify the primary attractors of an original network and a mutated network. When the primary attractors of the two networks were compared, the different states involved in the mutated network were ignored. As primary attractors can be cyclic attractors, which oscillate between more than two states, the averaged ratio of states of primary attractors of both the original network and every single node mutated network was calculated:

(5) $$B_{m}=\;\frac{\sum_{k}\frac{|\{s\;|s\in \left(A_{o}\cap A_{k}\right)\}|}{\left|\left\{s|s\in A_{o}\right\}\right|}}{2N}$$

*s* is a network state, and *A_o_* and *A_k_* are primary attractors of the original network and the mutated network, respectively. The robustness to perturbation is measured by the ratio of initial states converging to the same attractor independently of the single node perturbation. 2^10^ initial states were sampled and *N* perturbed states were generated for each initial state:

(6) $$B_{p}=\frac{\sum_{s\in S}\sum_{k=1}^{N}\theta \left(A_{s}=A_{s}^{k}\right)}{\left|S\right|}\;$$

S is a set of sampled initial states. *A*_s_ is an attractor reached from an initial state s whereas $A_{s}^{k}$ is an attractor state reached from a perturbed state of the initial state s by flipping the state of the *k*‐th node. *θ*(*l*) returns to 0 or 1 if *l* is false or true, respectively.

### Defining Alteration of Bladder Cancer Cell Lines and Cancer Patients

Molecular profiles of bladder cancer cell lines and cancer patients were obtained from the cBioPortal (http://www.cbioportal.org).^[^ ^64^, ^65^ ^]^ Among the data sets of cBioPortal, the Cancer Cell Line Encyclopedia data set was used for bladder cancer cell lines,^[^ ^66^ ^]^ published data of TCGA for bladder cancer patients,^[^ ^67^ ^]^ and TCGA Pan‐cancer atlas data for breast cancer patients.^[^ ^68^ ^]^ Alterations of genes are defined as i) genes with copy number alteration (CAN) and consistent mRNA expression (z‐scores relative to diploid samples, threshold ± 2.0) and ii) genes with a driver mutation while having no low mRNA expression. For the bladder cancer cell lines, alteration is defined as GOF alteration if the altered gene is an oncogene or LOF alteration if the altered gene is a tumor suppressor in OncoKB (https://www.oncokb.org).^[^ ^31^ ^]^ Kaplan‐Meier survival graphs were drawn by using the Python package “lifelines.”^[^ ^69^ ^]^

### Reagents and Antibodies

DMSO (Sigma‐Aldrich, D8418), TGF‐b1 (PeproTech, 100‐21), MK2206 (Selleck, S1078), and 5‐FU (Selleck, S1209) were used in this study. Anti‐phospho‐AKT (S473) antibody and anti‐AKT antibody were purchased from Cell Signaling Technology, Inc (Danvers, MA). Anti‐phopho‐Histone H2A.X(S139) antibody was obtained from Santa Cruz Biotechnology, Inc (Dallas, TX). The rabbit polyclonal anti‐GAPDH antibody was provided as a generous gift from Dr. Ki‐Sun Kwon (Korea Research Institute of Bioscience and Biotechnology).

### Cell Culture

The KU‐1919 cell line was generously provided by Dr. San‐Jin Lee (National Cancer Center Korea). KU‐1919 cells were cultured in RPMI 1640 (Welgene, Republic of Korea) supplemented with 10% fetal bovine serum (FBS, Welgene), 100 U mL^‐1^ penicillin, 100 µg mL^‐1^ streptomycin, and 0.25 µg mL^‐1^ Fungizone (Life Technologies, Carlsbad, CA). The HT‐1197 cell line was purchased from the Korea Cell Line Bank and cultivated in MEM (Welgene) supplemented with 10% FBS and 100 U mL^‐1^ penicillin, 100 µg mL^‐1^ streptomycin, and 0.25 µg mL^‐1^ Fungizone. All cells were cultured at 37 °C in a humidified 5% CO_2_ incubator.

### Small Interference RNA Knockdown

Cells were seeded at ≈50% confluence for siRNA transfection. Three different kinds of siRNAs targeting MAP3K1 were obtained from Bioneer and mixed with equal concentrations. AccuTarget Negative Control siRNA (Bioneer, Republic of Korea) was used as the control for each experiment. siRNAs were transfected with Lipofectamine RNAiMAX (Invitrogen, Waltham, MA) according to the manufacturer's instructions.

### Quantitative Reverse‐Transcriptase Polymerase Reaction

Messenger RNAs were extracted by using the easy‐spin Total RNA Extraction Kit (Intronbio, Republic of Korea). The synthesis of DNA from RNA templates was performed using a DiaStar RT Kit (Solgent, Republic of Korea) and 2X Taq premix (Solgent) according to the manufacturer's instructions. Amplification of the synthesized cDNA was reacted using SYBR Master Mix (GeNet Bio, Republic of Korea) and a QuantStudio5 qPCR machine (Applied Biosystems, Waltham, MA). The specific primer sequences were as follows:

MAP3K1 fwd, 5′‐CCAGACCAGTATCTCAGGAGATG‐3′;

MAP3K1 rev, 5′‐CCGCTAAACTGTGGCAAGGAGT‐3′;

GAPDH fwd, 5′‐TGATGACATCAAGAAGGTGGTGAAG‐3′;

GAPDH rev, 5′‐TCCTTGGAGGCCATGTGGGCCAT‐3′.

### Western Blot

Cells were lysed with lysis buffer (20 × 10^‐3^ m Hepes pH.7.2, 150 × 10^‐3^ m NaCl, 1% triton X‐100, 0.1% SDS, and 10% glycerol) containing protease and phosphatase inhibitor cocktail (Thermo Scientific, Waltham, MA). Cell lysates were mixed with 5X SDS‐ PAGE Loading buffer (LPS solution) and separated by sodium dodecyl sulfate‐polyacrylamide gel electrophoresis. Proteins of the lysates were transferred from the gel to 0.2 µL nitrocellulose membranes (Pall Corporation, NY). The membranes were then probed with primary antibodies and subsequently incubated with corresponding secondary antibodies conjugated with peroxidase. The blot of the membrane was detected through enhanced chemiluminescence (Thermo Fisher Scientific). Images were taken using a Fujifilm LAS‐3000 imager (Fujifilm, Japan).

### Crystal Violet Assay

Cells were rinsed once with 1× DPBS (Welgene) and fixed with 4% PFA (Sigma‐Aldrich). The fixed cells were stained with 1% crystal violet solution (Sigma‐Aldrich), and the stained cells were subsequently treated with 1% SDS (LPS Solution, Republic of Korea) solution in water. The optical density of the lysates was measured using a microplate reader (Bio‐Rad, Hercules, CA).

### Trypan Blue Assay

Floating cells and adherent cells were collected. Dispersed cells were stained with Trypan Blue Stain (0.4%, Gibco, Waltham, MA) and loaded on Cell Counting Slides (Bio‐Rad), and counted for viability using an Automated Cell Counter (Bio‐Rad).

### Immunofluorescence

HT‐1197 cells transfected with siRNAs were replated on culture glasses. Adherent cells on culture glasses were fixed with 4% PFA, which was followed by permeabilization with 0.1% Triton‐X‐100 (sigma‐aldrich). Fixed and permeabilized cells were incubated with primary antibodies for one hour followed by incubation with Goat anti‐Mouse secondary antibodies conjugated with Alexa Fluor 488 (Invitrogen) for 30 min at RT. The nuclei were stained with DAPI (4′,6‐diamidino‐2‐phenylindole, 1 µg mL^‐1^) for 5 min. Prolong gold antifade reagent (Invitrogen) was used to mount coverslips. The fluorescence of the cells was detected with a Zeiss Observer Z1 microscope equipped with Apotome 2 (Carl Zeiss, Germany). Image acquisition and processing were performed with AxioVision 4.8 (Carl Zeiss).

### Statistical Analysis

In the bladder cancer cell line experiments, statistically significant differences were determined by a two‐tailed Student's t‐test (*n* = 4). Logrank tests among two patient groups were done by using the function “logrank_test()” of the Python package “lifelines”^[^ ^69^ ^]^ for bladder cancer patients (*n* = 276). Linear regression was done by the function “lmplot” of the Python package “seaborn”^[^ ^70^, ^71^ ^]^ for random configuration models (*n* = 306).

## Conflict of Interest

The authors declare no conflict of interest.

## Author Contributions

Conceptualization: K.‐H.C., J.I.J., Methodology: J.I.J., Formal analysis: J.I.J., Investigation: J.I.J., H.‐J.P., Visualization: J.I.J., H.‐J.P., Supervision: K.‐H.C., Writing—original draft: K.‐H.C., J.I.J., H.‐J.P., Writing—review & editing: K.‐H.C.

## Supporting information

Supporting Information

Supplemental Table 1

Supplemental Table 2

## Data Availability

The data that support the findings of this study are available in the supplementary material of this article.

## References

[1] R. E. Pollack , H. Green , G. J. Todaro , Proc. Natl. Acad. Sci. USA 1968, 60, 126. 4297915 10.1073/pnas.60.1.126 PMC539091

[2] M. Tuynder , L. Susini , S. Prieur , S. Besse , G. Fiucci , R. Amson , A. Telerman , Proc. Natl. Acad. Sci. USA 2002, 99, 14976. 12399545 10.1073/pnas.222470799 PMC137530

[3] M. Tuynder , G. Fiucci , S. Prieur , A. Lespagnol , A. Geant , S. Beaucourt , D. Duflaut , S. Besse , L. Susini , J. Cavarelli , D. Moras , R. Amson , A. Telerman , Proc. Natl. Acad. Sci. USA 2004, 101, 15364. 15489264 10.1073/pnas.0406776101 PMC523462

[4] L. E. Dow , K. P. O'Rourke , J. Simon , D. F. Tschaharganeh , J. H. van Es , H. Clevers , S. W. Lowe , Cell 2015, 161, 1539. 26091037 10.1016/j.cell.2015.05.033 PMC4475279

[5] K.‐H. Cho , S. Lee , D. Kim , D. Shin , J. I. Joo , S.‐M. Park , Curr. Opin. Syst. Biol. 2017, 2, 49.

[6] S. N. Sreenath , K. H. Cho , P. Wellstead , Essays Biochem. 2008, 45, 1. 18793120 10.1042/BSE0450001

[7] S. H. Cho , S. M. Park , H. S. Lee , H. Y. Lee , K. H. Cho , BMC Syst. Biol. 2016, 10, 96. 27765040 10.1186/s12918-016-0341-9 PMC5072344

[8] S. R. Choi , C. Y. Hwang , J. Lee , K. H. Cho , Cancer Res. 2022, 82, 320. 34845001 10.1158/0008-5472.CAN-21-0621

[9] M. Choi , J. Shi , Y. Zhu , R. Yang , K. H. Cho , Nat. Commun. 2017, 8, 1940. 29208897 10.1038/s41467-017-02160-5 PMC5717260

[10] J. I. Joo , J. X. Zhou , S. Huang , K. H. Cho , Sci. Rep. 2018, 8, 12077. 30104572 10.1038/s41598-018-30544-0 PMC6089891

[11] J. Kim , S. M. Park , K. H. Cho , Sci. Rep. 2013, 3, 2223. 23860463 10.1038/srep02223 PMC3713565

[12] S. M. Choo , B. Ban , J. I. Joo , K. H. Cho , BMC Syst. Biol. 2018, 12, 49. 29622038 10.1186/s12918-018-0576-8 PMC5887232

[13] J. G. Zanudo , R. Albert , PLoS Comput. Biol. 2015, 11, e1004193. 25849586 10.1371/journal.pcbi.1004193 PMC4388852

[14] G. Yang , J. Gomez Tejeda Zanudo , R. Albert , Front. Physiol. 2018, 9, 454. 29867523 10.3389/fphys.2018.00454 PMC5951947

[15] T. Akutsu , S. Kosub , A. A. Melkman , T. Tamura , IEEE/ACM Trans. Comput. Biol. Bioinf. 2012, 9, 1410. 10.1109/TCBB.2012.87 22689081

[16] S. Y. Shin , T. Kim , H. S. Lee , J. H. Kang , J. Y. Lee , K. H. Cho , D. H. Kim , Nat. Commun. 2014, 5, 5777. 25517116 10.1038/ncomms6777 PMC4284638

[17] T. Helikar , B. Kowal , J. A. Rogers , Clin. Pharmacol. Ther. 2013, 93, 393. 23549147 10.1038/clpt.2013.41 PMC5242230

[18] V. L. Chudasama , M. A. Ovacik , D. R. Abernethy , D. E. Mager , J. Pharmacol. Exp. Ther. 2015, 354, 448. 26163548 10.1124/jpet.115.224766 PMC4538876

[19] Z. Mai , H. Liu , J. Theor. Biol. 2009, 259, 760. 19422837 10.1016/j.jtbi.2009.04.024

[20] S. von der Heyde , C. Bender , F. Henjes , J. Sonntag , U. Korf , T. Beissbarth , BMC Syst. Biol. 2014, 8, 75. 24970389 10.1186/1752-0509-8-75 PMC4087127

[21] J. Lu , H. Zeng , Z. Liang , L. Chen , L. Zhang , H. Zhang , H. Liu , H. Jiang , B. Shen , M. Huang , M. Geng , S. Spiegel , C. Luo , Sci. Rep. 2015, 5, 14739. 26446703 10.1038/srep14739 PMC4597205

[22] L. Calzone , L. Tournier , S. Fourquet , D. Thieffry , B. Zhivotovsky , E. Barillot , A. Zinovyev , PLoS Comput. Biol. 2010, 6, e1000702. 20221256 10.1371/journal.pcbi.1000702 PMC2832675

[23] A. Singh , J. M. Nascimento , S. Kowar , H. Busch , M. Boerries , Bioinformatics 2012, 28, i495. 22962472 10.1093/bioinformatics/bts410 PMC3436837

[24] O. Sahin , H. Frohlich , C. Lobke , U. Korf , S. Burmester , M. Majety , J. Mattern , I. Schupp , C. Chaouiya , D. Thieffry , A. Poustka , S. Wiemann , T. Beissbarth , D. Arlt , BMC Syst. Biol. 2009, 3, 1. 19118495 10.1186/1752-0509-3-1 PMC2652436

[25] L. Grieco , L. Calzone , I. Bernard‐Pierrot , F. Radvanyi , B. Kahn‐Perles , D. Thieffry , PLoS Comput. Biol. 2013, 9, e1003286. 24250280 10.1371/journal.pcbi.1003286 PMC3821540

[26] S. Sridharan , R. Layek , A. Datta , J. Venkatraj , BMC Genomics 2012, 13, S4. 10.1186/1471-2164-13-S6-S4 PMC3481480 23134720

[27] T. Helikar , J. Konvalina , J. Heidel , J. A. Rogers , Proc. Natl. Acad. Sci. USA 2008, 105, 1913. 18250321 10.1073/pnas.0705088105 PMC2538858

[28] R. Zhang , M. V. Shah , J. Yang , S. B. Nyland , X. Liu , J. K. Yun , R. Albert , T. P. Loughran Jr , Proc. Natl. Acad. Sci. USA 2008, 105, 16308. 18852469 10.1073/pnas.0806447105 PMC2571012

[29] A. Saadatpour , R. S. Wang , A. Liao , X. Liu , T. P. Loughran , I. Albert , R. Albert , PLoS Comput. Biol. 2011, 7, e1002267. 22102804 10.1371/journal.pcbi.1002267 PMC3213185

[30] D. P. Cohen , L. Martignetti , S. Robine , E. Barillot , A. Zinovyev , L. Calzone , PLoS Comput. Biol. 2015, 11, e1004571. 26528548 10.1371/journal.pcbi.1004571 PMC4631357

[31] D. Chakravarty , J. Gao , S. M. Phillips , R. Kundra , H. Zhang , J. Wang , J. E. Rudolph , R. Yaeger , T. Soumerai , M. H. Nissan , M. T. Chang , S. Chandarlapaty , T. A. Traina , P. K. Paik , A. L. Ho , F. M. Hantash , A. Grupe , S. S. Baxi , M. K. Callahan , A. Snyder , P. Chi , D. Danila , M. Gounder , J. J. Harding , M. D. Hellmann , G. Iyer , Y. Janjigian , T. Kaley , D. A. Levine , M. Lowery , et al., JCO Precis. Oncol. 2017, 1, 1.

[32] K. Kahata , M. S. Dadras , A. Moustakas , Cold Spring Harbor Perspect. Biol. 2018, 10, a022194. 10.1101/cshperspect.a022194 PMC5749157 28246184

[33] V. Syed , J. Cell. Biochem. 2016, 117, 1279. 26774024 10.1002/jcb.25496

[34] A. Sharma , K. Singh , A. Almasan , Methods Mol. Biol. 2012, 920, 613. 22941631 10.1007/978-1-61779-998-3_40

[35] M. Aldana , E. Balleza , S. Kauffman , O. Resendiz , J. Theor. Biol. 2007, 245, 433. 17188715 10.1016/j.jtbi.2006.10.027

[36] J. Kim , D. Vandamme , J. R. Kim , A. G. Munoz , W. Kolch , K. H. Cho , PLoS Comput. Biol. 2014, 10, e1003763. 25077791 10.1371/journal.pcbi.1003763 PMC4117429

[37] S. Ciliberti , O. C. Martin , A. Wagner , Proc. Natl. Acad. Sci. USA 2007, 104, 13591. 17690244 10.1073/pnas.0705396104 PMC1959426

[38] A. Wagner , FEBS Lett. 2005, 579, 1772. 15763550 10.1016/j.febslet.2005.01.063

[39] C. H. Seo , J. R. Kim , M. S. Kim , K. H. Cho , Bioinformatics 2009, 25, 1898. 19439566 10.1093/bioinformatics/btp316

[40] F. Li , T. Long , Y. Lu , Q. Ouyang , C. Tang , Proc. Natl. Acad. Sci. USA 2004, 101, 4781. 15037758 10.1073/pnas.0305937101 PMC387325

[41] I. Martincorena , A. Roshan , M. Gerstung , P. Ellis , P. Van Loo , S. McLaren , D. C. Wedge , A. Fullam , L. B. Alexandrov , J. M. Tubio , L. Stebbings , A. Menzies , S. Widaa , M. R. Stratton , P. H. Jones , P. J. Campbell , Science 2015, 348, 880. 25999502 10.1126/science.aaa6806 PMC4471149

[42] I. Martincorena , J. C. Fowler , A. Wabik , A. R. J. Lawson , F. Abascal , M. W. J. Hall , A. Cagan , K. Murai , K. Mahbubani , M. R. Stratton , R. C. Fitzgerald , P. A. Handford , P. J. Campbell , K. Saeb‐Parsy , P. H. Jones , Science 2018, 362, 911. 30337457 10.1126/science.aau3879 PMC6298579

[43] S. Lee , C. Lee , C. Y. Hwang , D. Kim , Y. Han , S. N. Hong , S. H. Kim , K. H. Cho , Mol. Cancer Res. 2020, 18, 118. 31896605 10.1158/1541-7786.MCR-19-0450

[44] Z. Cheng , Z. He , Y. Cai , C. Zhang , G. Fu , H. Li , W. Sun , C. Liu , X. Cui , B. Ning , D. Xiang , T. Zhou , X. Li , W. Xie , H. Wang , J. Ding , Cell Res. 2019, 29, 124. 30560924 10.1038/s41422-018-0111-x PMC6355772

[45] M. Boia‐Ferreira , A. B. Basilio , A. E. Hamasaki , F. H. Matsubara , M. H. Appel , C. R. V. Da Costa , R. Amson , A. Telerman , O. M. Chaim , S. S. Veiga , A. Senff‐Ribeiro , Br. J. Cancer 2017, 117, 656. 28751755 10.1038/bjc.2017.230 PMC5572181

[46] A. Vinayagam , T. E. Gibson , H. J. Lee , B. Yilmazel , C. Roesel , Y. Hu , Y. Kwon , A. Sharma , Y. Y. Liu , N. Perrimon , A. L. Barabasi , Proc. Natl. Acad. Sci. USA 2016, 113, 4976. 27091990 10.1073/pnas.1603992113 PMC4983807

[47] F. K. Hamey , S. Nestorowa , S. J. Kinston , D. G. Kent , N. K. Wilson , B. Gottgens , Proc. Natl. Acad. Sci. USA 2017, 114, 5822. 28584094 10.1073/pnas.1610609114 PMC5468644

[48] T. Heydari , M. A. Langley , C. L. Fisher , D. Aguilar‐Hidalgo , S. Shukla , A. Yachie‐Kinoshita , M. Hughes , K. M. McNagny , P. W. Zandstra , PLoS Comput. Biol. 2022, 18, e1009907. 35213533 10.1371/journal.pcbi.1009907 PMC8906617

[49] A. Pratapa , A. P. Jalihal , J. N. Law , A. Bharadwaj , T. M. Murali , Nat. Methods 2020, 17, 147. 31907445 10.1038/s41592-019-0690-6 PMC7098173

[50] V. Moignard , S. Woodhouse , L. Haghverdi , A. J. Lilly , Y. Tanaka , A. C. Wilkinson , F. Buettner , I. C. Macaulay , W. Jawaid , E. Diamanti , S. I. Nishikawa , N. Piterman , V. Kouskoff , F. J. Theis , J. Fisher , B. Gottgens , Nat. Biotechnol. 2015, 33, 269. 25664528 10.1038/nbt.3154 PMC4374163

[51] I. Martincorena , K. M. Raine , M. Gerstung , K. J. Dawson , K. Haase , P. Van Loo , H. Davies , M. R. Stratton , P. J. Campbell , Cell 2018, 173, 1823. 10.1016/j.cell.2018.06.001 PMC6005233 29906452

[52] S. W. Lowe , H. E. Ruley , T. Jacks , D. E. Housman , Cell 1993, 74, 957. 8402885 10.1016/0092-8674(93)90719-7

[53] M. J. Lee , A. S. Ye , A. K. Gardino , A. M. Heijink , P. K. Sorger , G. MacBeath , M. B. Yaffe , Cell 2012, 149, 780. 22579283 10.1016/j.cell.2012.03.031 PMC3501264

[54] N. Coleman , J. T. Moyers , A. Harbery , I. Vivanco , T. A. Yap , Pharmgenomics Pers. Med. 2021, 14, 1517. 34858045 10.2147/PGPM.S305068 PMC8630372

[55] S. B. Kim , R. Dent , S. A. Im , M. Espie , S. Blau , A. R. Tan , S. J. Isakoff , M. Oliveira , C. Saura , M. J. Wongchenko , A. V. Kapp , W. Y. Chan , S. M. Singel , D. J. Maslyar , J. Baselga , L. investigators , Lancet Oncol. 2017, 18, 1360. 28800861 10.1016/S1470-2045(17)30450-3 PMC5626630

[56] T. T. Pham , S. P. Angus , G. L. Johnson , Genes Cancer 2013, 4, 419. 24386504 10.1177/1947601913513950 PMC3877667

[57] K. Nguyen , M. N. Tran , A. Rivera , T. Cheng , G. O. Windsor , A. B. Chabot , J. E. Cavanaugh , B. M. Collins‐Burow , S. B. Lee , D. H. Drewry , P. T. Flaherty , M. E. Burow , Front. Biosci. 2022, 27, 167. 10.31083/j.fbl2705167 35638434

[58] H. S. Lee , C. Y. Hwang , S. Y. Shin , K. S. Kwon , K. H. Cho , Sci. Signaling 2014, 7, ra52. 10.1126/scisignal.2005260 24894995

[59] A. Telerman , R. Amson , in TCTP/tpt1 ‐ Remodeling Signaling from Stem Cell to Disease, Results and Problems in Cell Differentiation (Eds: A. Telerman , R. Amson ), Vol. 64, Springer, Berlin 2017, p. 1.

[60] R. Amson , S. Pece , A. Lespagnol , R. Vyas , G. Mazzarol , D. Tosoni , I. Colaluca , G. Viale , S. Rodrigues‐Ferreira , J. Wynendaele , O. Chaloin , J. Hoebeke , J. C. Marine , P. P. Di Fiore , A. Telerman , Nat. Med. 2011, 18, 91. 22157679 10.1038/nm.2546

[61] S. A. Kauffman , J. Theor. Biol. 1969, 22, 437. 5803332 10.1016/0022-5193(69)90015-0

[62] M. E. Newman , S. H. Strogatz , D. J. Watts , Phys. Rev. E: Stat., Nonlinear, Soft Matter Phys. 2001, 64, 026118. 10.1103/PhysRevE.64.026118 11497662

[63] I. Shmulevich , S. A. Kauffman , Phys. Rev. Lett. 2004, 93, 048701. 15323803 10.1103/PhysRevLett.93.048701 PMC1490311

[64] E. Cerami , J. Gao , U. Dogrusoz , B. E. Gross , S. O. Sumer , B. A. Aksoy , A. Jacobsen , C. J. Byrne , M. L. Heuer , E. Larsson , Y. Antipin , B. Reva , A. P. Goldberg , C. Sander , N. Schultz , Cancer Discovery 2012, 2, 401. 22588877 10.1158/2159-8290.CD-12-0095 PMC3956037

[65] J. Gao , B. A. Aksoy , U. Dogrusoz , G. Dresdner , B. Gross , S. O. Sumer , Y. Sun , A. Jacobsen , R. Sinha , E. Larsson , E. Cerami , C. Sander , N. Schultz , Sci. Signaling 2013, 6, pl1. 10.1126/scisignal.2004088 PMC4160307 23550210

[66] M. Ghandi , F. W. Huang , J. Jane‐Valbuena , G. V. Kryukov , C. C. Lo , E. R. McDonald 3rd , J. Barretina , E. T. Gelfand , C. M. Bielski , H. Li , K. Hu , A. Y. Andreev‐Drakhlin , J. Kim , J. M. Hess , B. J. Haas , F. Aguet , B. A. Weir , M. V. Rothberg , B. R. Paolella , M. S. Lawrence , R. Akbani , Y. Lu , H. L. Tiv , P. C. Gokhale , A. de Weck , A. A. Mansour , C. Oh , J. Shih , K. Hadi , Y. Rosen , et al., Nature 2019, 569, 503. 31068700 10.1038/s41586-019-1186-3 PMC6697103

[67] A. G. Robertson , J. Kim , H. Al‐Ahmadie , J. Bellmunt , G. Guo , A. D. Cherniack , T. Hinoue , P. W. Laird , K. A. Hoadley , R. Akbani , M. A. A. Castro , E. A. Gibb , R. S. Kanchi , D. A. Gordenin , S. A. Shukla , F. Sanchez‐Vega , D. E. Hansel , B. A. Czerniak , V. E. Reuter , X. Su , B. de Sa Carvalho , V. S. Chagas , K. L. Mungall , S. Sadeghi , C. S. Pedamallu , Y. Lu , L. J. Klimczak , J. Zhang , C. Choo , A. I. Ojesina , et al., Cell 2018, 174, 1033. 30096301 10.1016/j.cell.2018.07.036 PMC6297116

[68] K. A. Hoadley , C. Yau , T. Hinoue , D. M. Wolf , A. J. Lazar , E. Drill , R. Shen , A. M. Taylor , A. D. Cherniack , V. Thorsson , R. Akbani , R. Bowlby , C. K. Wong , M. Wiznerowicz , F. Sanchez‐Vega , A. G. Robertson , B. G. Schneider , M. S. Lawrence , H. Noushmehr , T. M. Malta , N. Cancer Genome Atlas , J. M. Stuart , C. C. Benz , P. W. Laird , Cell 2018, 173, 291.29625048

[69] C. Davidson‐Pilon , J. Open Source Software 2019, 4, 1317.

[70] M. L. Waskom , J. Open Source Software 2021, 6, 3021.

[71] J. D. Hunter , Comput. Sci. Eng. 2007, 9, 90.

