# Supplementary material for: Normalizing Input–Output Relationships of Cancer Networks for Reversion Therapy
Source: Adv Sci (Weinh). 2023 Jun 2;10(24):2207322. doi: 10.1002/advs.202207322 (PMC10460890; doi:10.1002/advs.202207322)
Supplement: Supplementary file 1 — Supporting Information [file ADVS-10-2207322-s001.pdf]

ADVANCED SCIENCE
Open Access

Supporting Information

for *Adv. Sci.*, DOI 10.1002/advs.202207322

Normalizing Input–Output Relationships of Cancer Networks for Reversion Therapy

*Jae Il Joo, Hwa-Jeong Park and Kwang-Hyun Cho**

Supporting Information

Normalizing input-output relationships of cancer networks for reversion therapy

*Jae Il Joo, Hwa-Jeong Park, and Kwang-Hyun Cho**

This file includes:

Supplementary Figure S1 to S10

Other Supplementary Materials for this manuscript include the following:

Supplementary Table S1 to S2

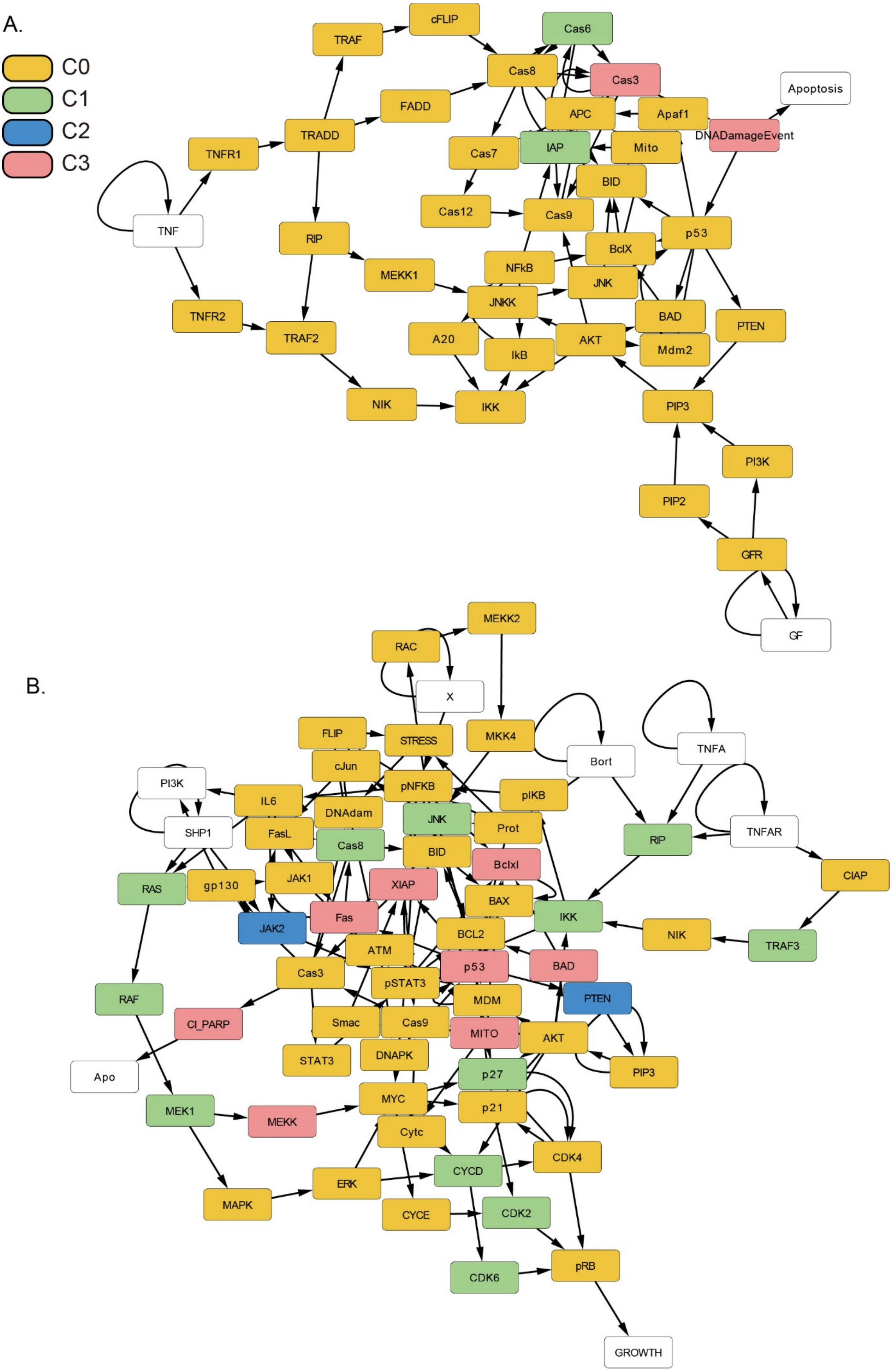
A.
C0
C1
C2
C3
cFLIP
Cas6
TRAF
Cas8
Cas3
Apoptosis
FADD
APC
Apaf1
TRADD
TNFR1
Cas7
IAP
Mito
DNADamageEvent
BID
TNF
Cas12
Cas9
p53
RIP
BclX
NFkB
MEKK1
JNK
JNKK
BAD
PTEN
TNFR2
TRAF2
A20
AKT
IkB
Mdm2
NIK
IKK
PIP3
PI3K
PIP2
GFR
GF
B.
MEKK2
RAC
X
FLIP
STRESS
MKK4
Bort
TNFA
cJun
PI3K
IL6
pNFKB
pIKB
DNAdam
SHP1
FasL
JNK
Prot
RIP
TNFAR
Cas8
BID
Bclxl
RAS
gp130
JAK1
XIAP
BAX
CIAP
IKK
JAK2
Fas
BCL2
NIK
TRAF3
ATM
p53
BAD
Cas3
pSTAT3
MDM
PTEN
RAF
CI_PARP
Smac
Cas9
MITO
AKT
STAT3
DNAPK
Apo
p27
PIP3
MEK1
MEKK
MYC
p21
Cytc
CDK4
ERK
CYCD
MAPK
CYCE
CDK2
CDK6
pRB
GROWTH

C.

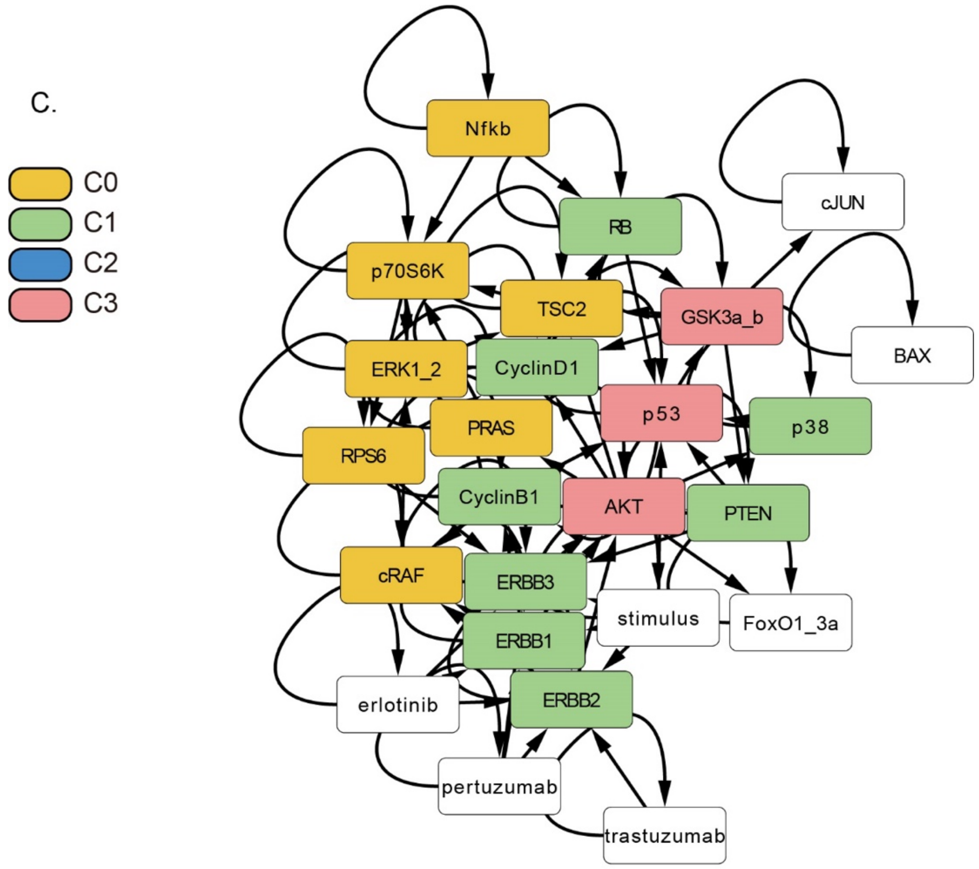
C0
C1
C2
C3
Nfkb
cJUN
RB
p70S6K
TSC2
GSK3a_b
BAX
ERK1_2
CyclinD1
p53
p38
PRAS
RPS6
CyclinB1
AKT
PTEN
cRAF
ERBB3
stimulus
FoxO1_3a
ERBB1
erlotinib
ERBB2
pertuzumab
trastuzumab

D.

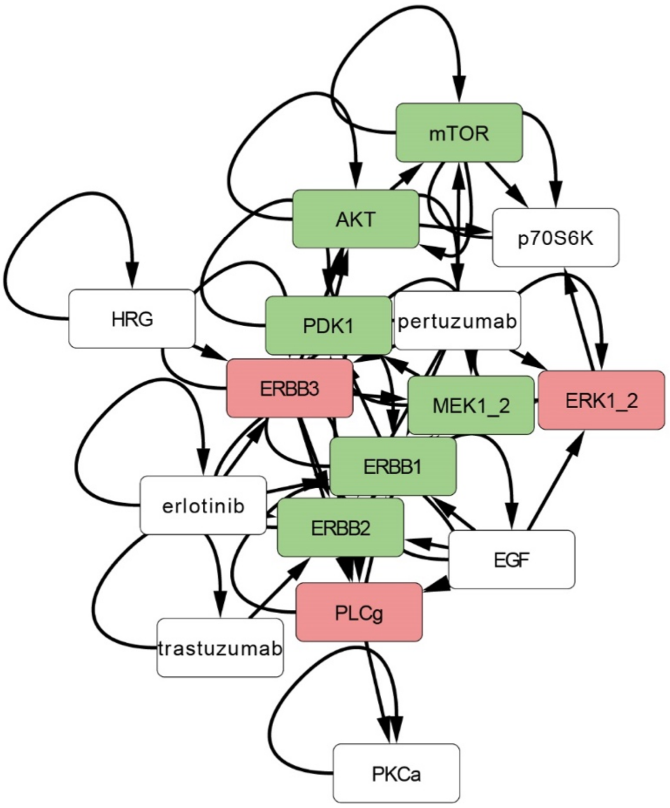
mTOR
AKT
p70S6K
HRG
PDK1
pertuzumab
ERBB3
MEK1_2
ERK1_2
ERBB1
erlotinib
ERBB2
EGF
PLCg
trastuzumab
PKCa

E.

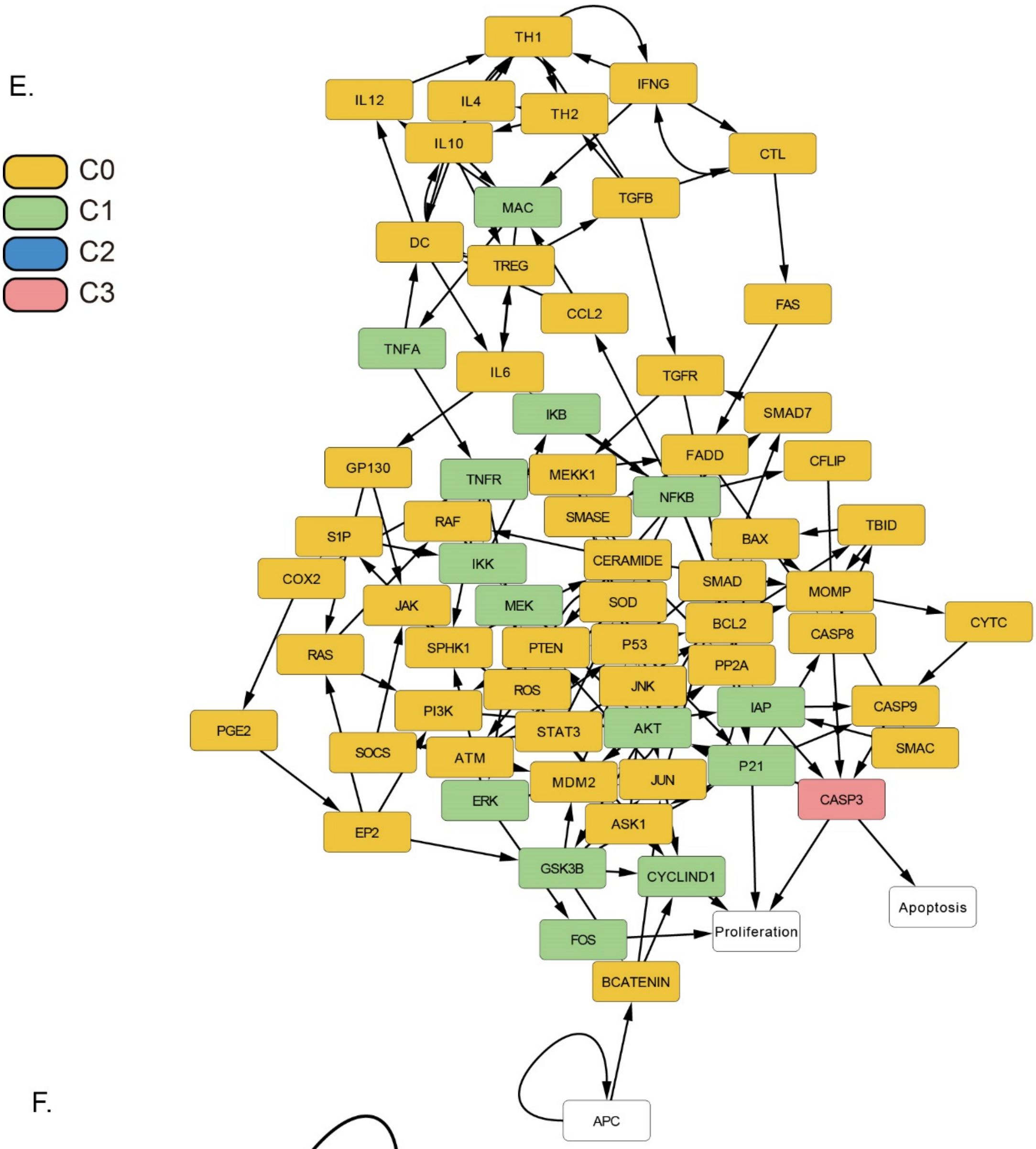
C0
C1
C2
C3
TH1
IL12
IL4
TH2
IFNG
IL10
CTL
MAC
TGFB
DC
TREG
CCL2
FAS
TNFA
IL6
TGFR
IKB
SMAD7
GP130
TNFR
MEKK1
FADD
CFLIP
NFKB
RAF
SMASE
S1P
TBID
BAX
IKK
CERAMIDE
COX2
SMAD
MOMP
JAK
MEK
SOD
CYTC
BCL2
CASP8
RAS
SPHK1
PTEN
P53
PP2A
ROS
JNK
PI3K
IAP
CASP9
PGE2
STAT3
AKT
SOCS
ATM
SMAC
P21
MDM2
JUN
CASP3
ERK
ASK1
EP2
GSK3B
CYCLIND1
Apoptosis
Proliferation
FOS
BCATENIN
APC

F.

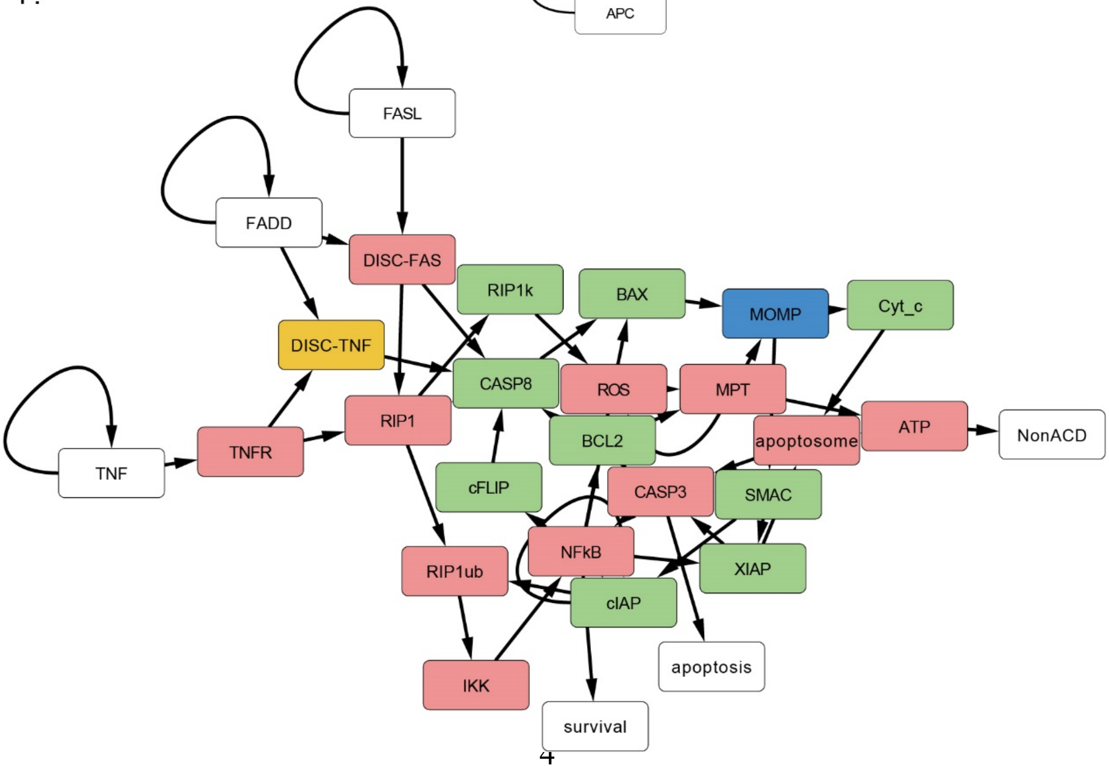
FASL
FADD
DISC-FAS
RIP1k
BAX
MOMP
Cyt_c
DISC-TNF
CASP8
ROS
MPT
RIP1
BCL2
apoptosome
ATP
NonACD
TNF
TNFR
cFLIP
CASP3
SMAC
NFkB
XIAP
RIP1ub
cIAP
IKK
apoptosis
survival

G.

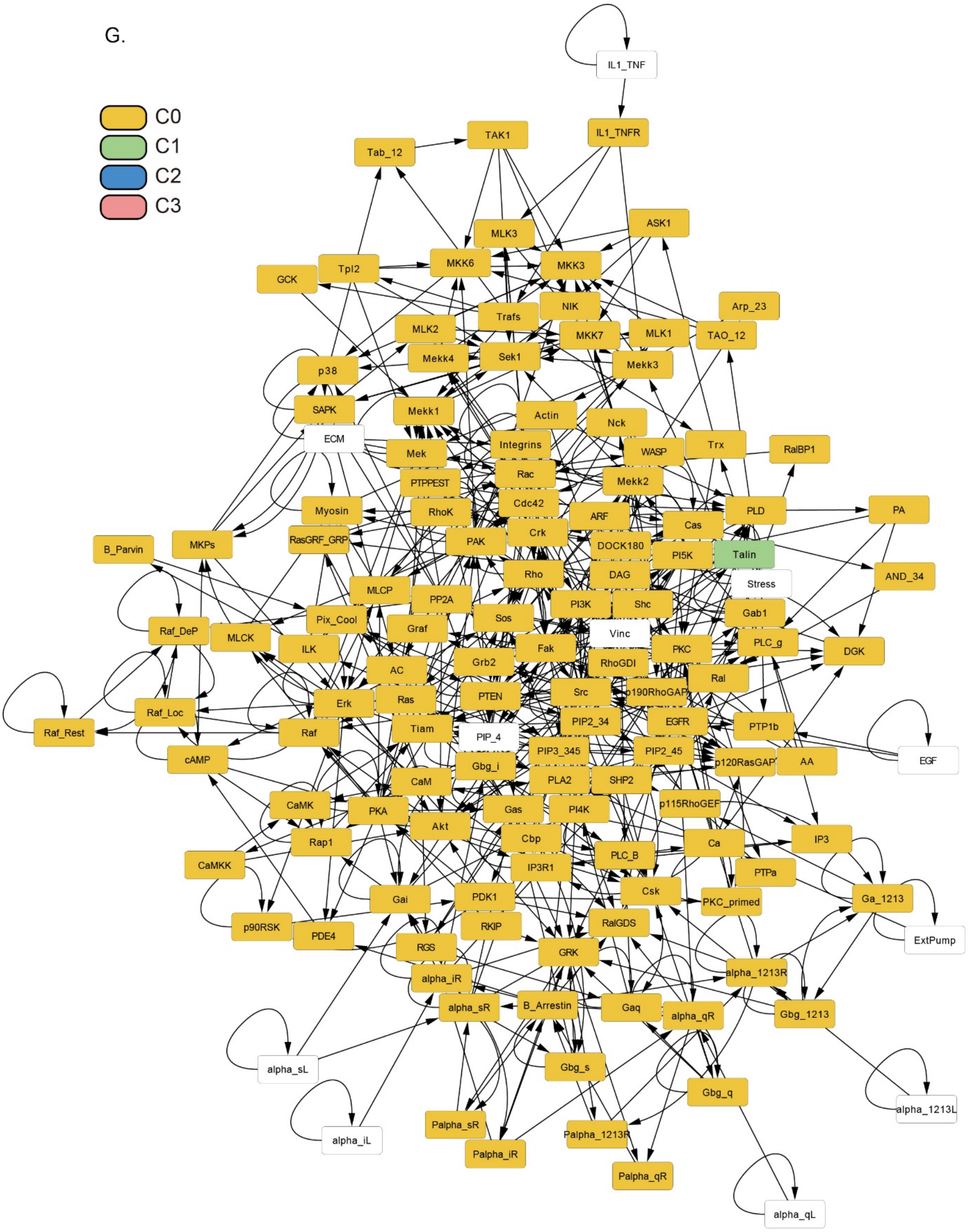
C0
C1
C2
C3
IL1_TNF
IL1_TNFR
TAK1
Tab_12
ASK1
MLK3
MKK6
MKK3
GCK
Tpl2
Arp_23
NIK
Trafs
MLK2
MKK7
MLK1
TAO_12
Mekk4
Sek1
Mekk3
p38
SAPK
Mekk1
Actin
Nck
ECM
Mek
Integrins
WASP
Trx
RalBP1
PTPPEST
Rac
Mekk2
Cdc42
Myosin
RhoK
ARF
Cas
PLD
PA
RasGRF_GRP
PAK
Crk
DOCK180
PI5K
Talin
B_Parvin
MKPs
Rho
DAG
Stress
AND_34
MLCP
PP2A
PI3K
Shc
Gab1
Pix_Cool
Sos
Graf
Vinc
Raf_DeP
MLCK
ILK
Fak
PKC
PLC_g
DGK
AC
Grb2
RhoGDI
Ral
Erk
Ras
PTEN
Src
p190RhoGAP
Raf_Loc
Tiam
PIP2_34
EGFR
PTP1b
Raf_Rest
Raf
PIP_4
cAMP
Gbg_i
PIP3_345
PIP2_45
p120RasGAP
AA
EGF
CaM
PLA2
SHP2
CaMK
PKA
Gas
PI4K
p115RhoGEF
Akt
Cbp
Rap1
Ca
IP3
PLC_B
CaMKK
IP3R1
PTPa
Csk
Ga_1213
PDK1
Gai
PKC_primed
p90RSK
RKIP
RalGDS
PDE4
ExtPump
RGS
GRK
alpha_1213R
alpha_iR
B_Arrestin
alpha_sR
Gaq
alpha_qR
Gbg_1213
Gbg_s
alpha_sL
Gbg_q
alpha_1213L
Palpha_sR
Palpha_1213R
alpha_iL
Palpha_iR
Palpha_qR
alpha_qL

H.

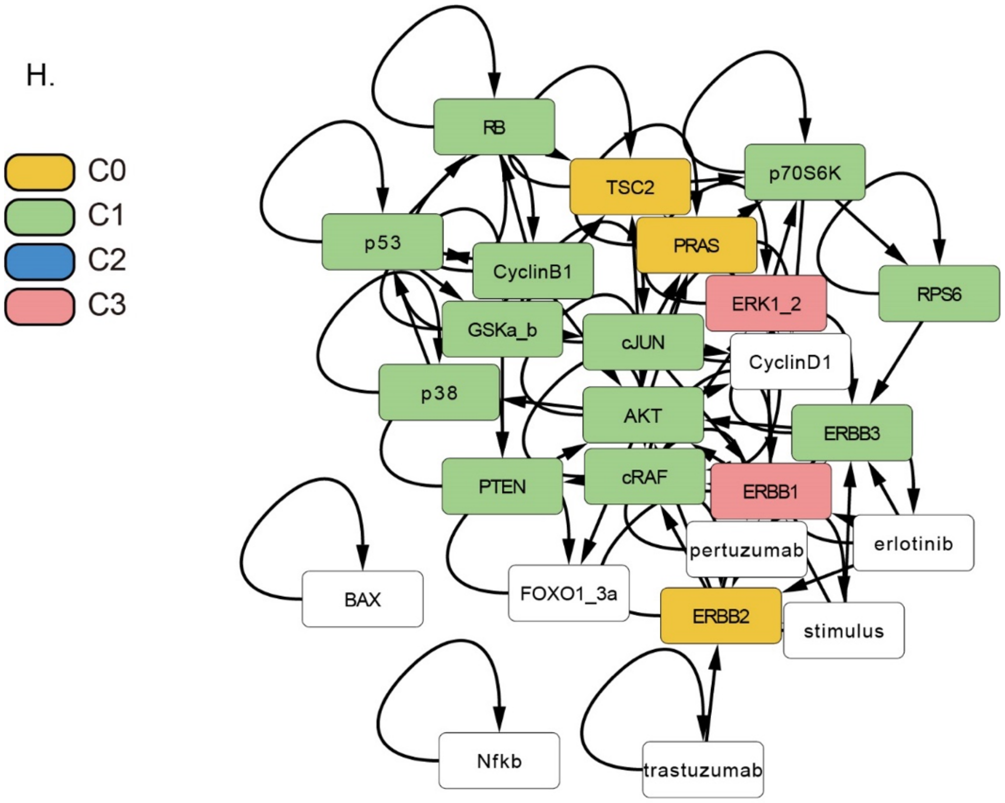
C0
C1
C2
C3
RB
TSC2
p70S6K
p53
PRAS
CyclinB1
ERK1_2
RPS6
GSKa_b
cJUN
CyclinD1
p38
AKT
ERBB3
PTEN
cRAF
ERBB1
pertuzumab
erlotinib
BAX
FOXO1_3a
ERBB2
stimulus
Nfkb
trastuzumab

I.

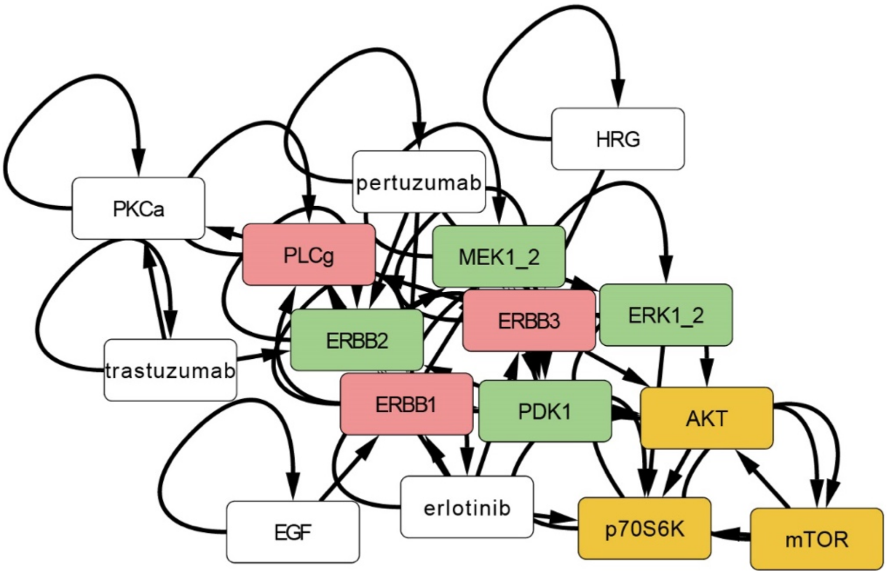
HRG
PKCa
pertuzumab
PLCg
MEK1_2
ERBB3
ERK1_2
trastuzumab
ERBB2
ERBB1
PDK1
AKT
EGF
erlotinib
p70S6K
mTOR

J.

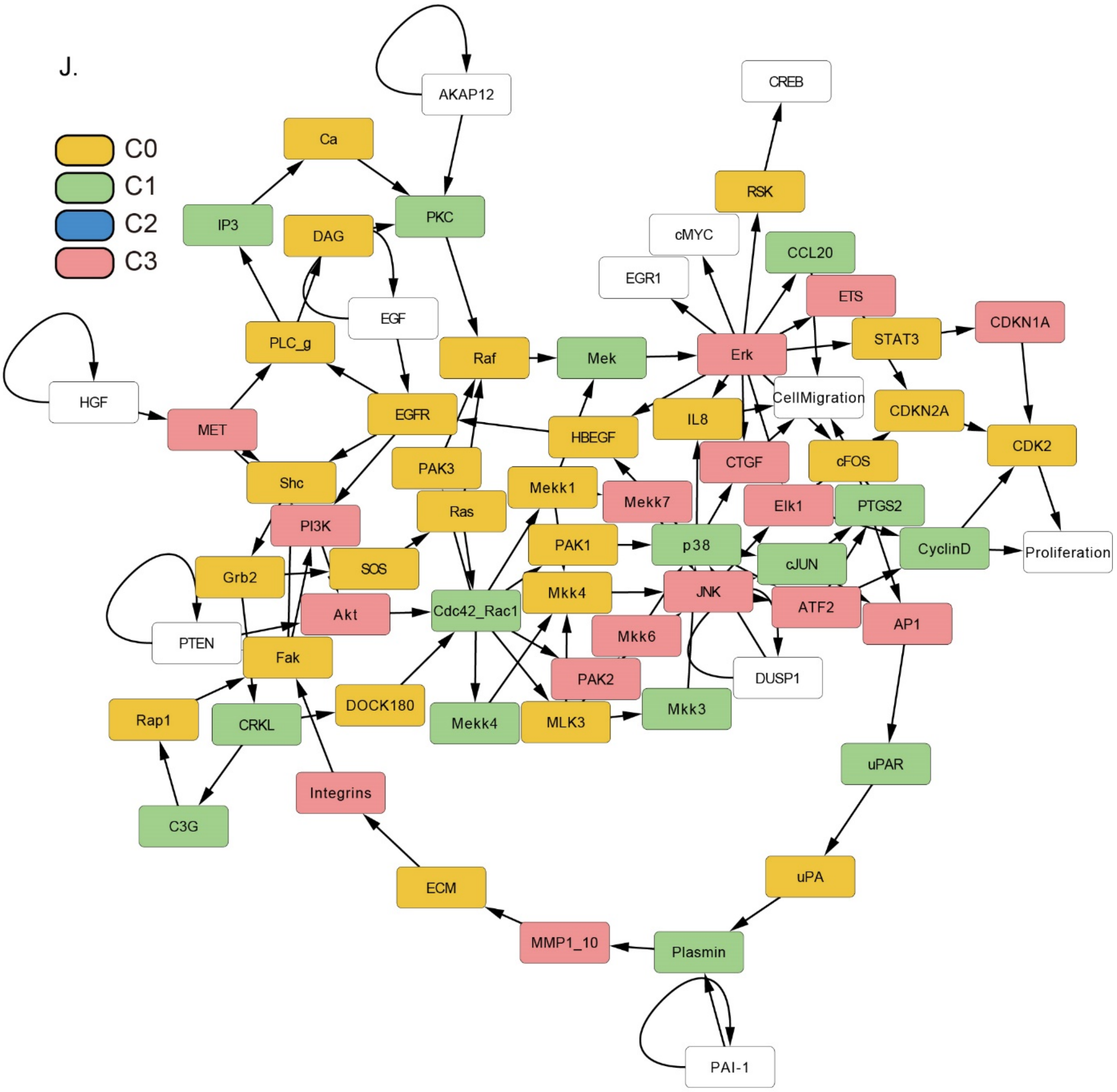
C0
C1
C2
C3
AKAP12
Ca
PKC
IP3
DAG
EGF
PLC_g
HGF
MET
EGFR
Raf
Mek
Erk
CREB
RSK
cMYC
EGR1
CCL20
ETS
STAT3
CDKN1A
CellMigration
CDKN2A
CDK2
IL8
HBEGF
CTGF
cFOS
Shc
PAK3
Mekk1
Mekk7
PI3K
Ras
Elk1
PTGS2
PAK1
p38
CyclinD
Proliferation
Grb2
SOS
cJUN
Mkk4
JNK
ATF2
AP1
PTEN
Akt
Cdc42_Rac1
Mkk6
Fak
DUSP1
PAK2
DOCK180
Mkk3
Rap1
CRKL
Mekk4
MLK3
uPAR
Integrins
C3G
uPA
ECM
MMP1_10
Plasmin
PAI-1

K.

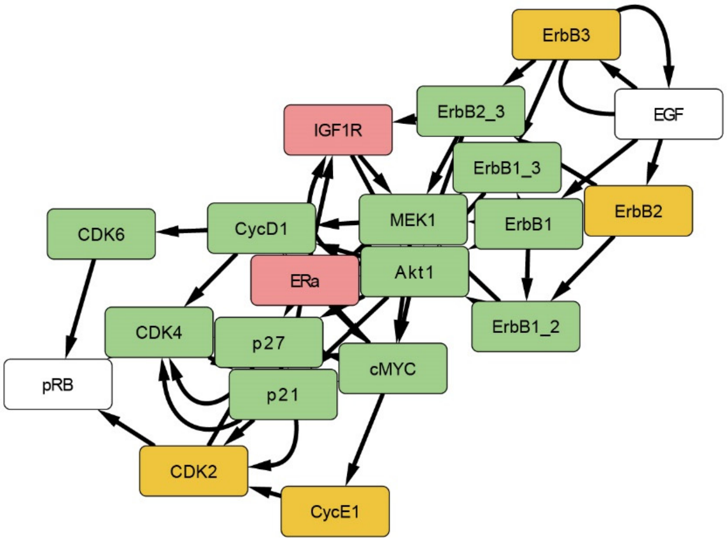
ErbB3
EGF
ErbB2_3
IGF1R
ErbB1_3
ErbB2
CDK6
CycD1
MEK1
ErbB1
ERa
Akt1
ErbB1_2
CDK4
p27
cMYC
pRB
p21
CDK2
CycE1

L.

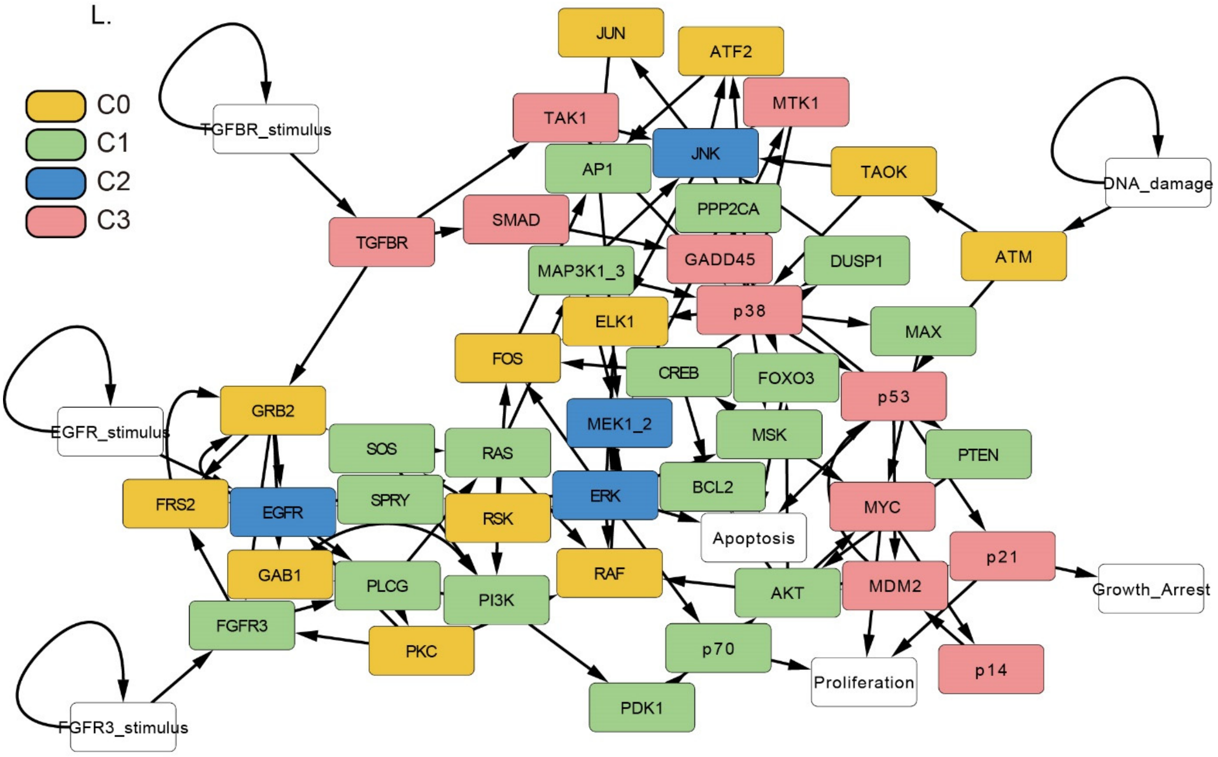
C0
C1
C2
C3
TGFBR_stimulus
JUN
ATF2
TAK1
MTK1
JNK
AP1
TAOK
DNA_damage
SMAD
PPP2CA
TGFBR
GADD45
DUSP1
ATM
MAP3K1_3
p38
ELK1
MAX
FOS
CREB
FOXO3
p53
GRB2
EGFR_stimulus
SOS
RAS
MEK1_2
MSK
PTEN
BCL2
SPRY
ERK
MYC
FRS2
EGFR
RSK
Apoptosis
GAB1
PLCG
RAF
p21
AKT
MDM2
Growth_Arrest
PI3K
FGFR3
PKC
p70
p14
Proliferation
PDK1
FGFR3_stimulus

M.

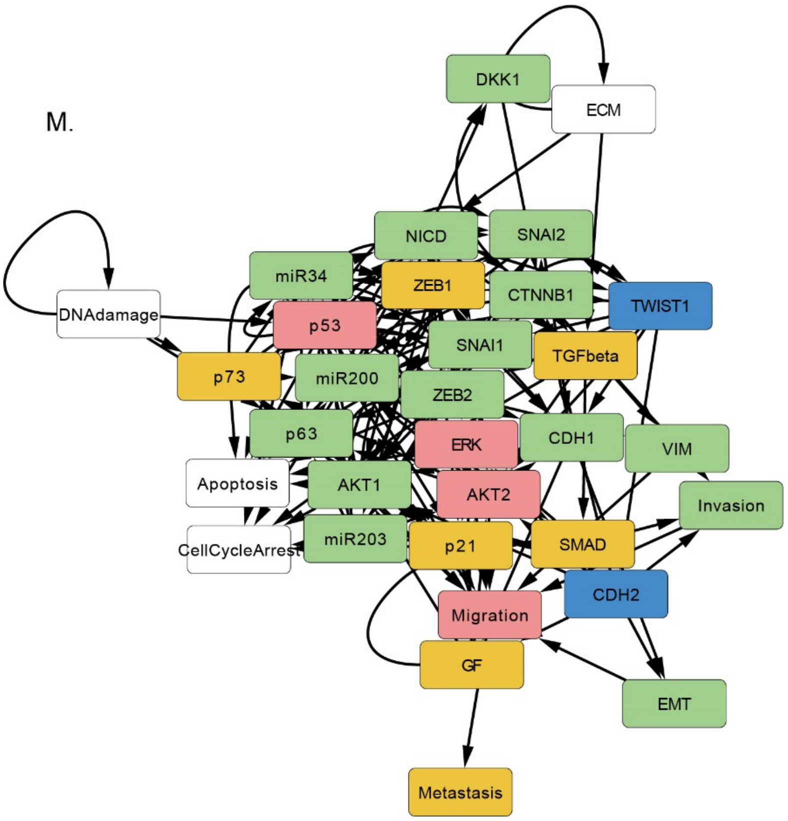
DKK1
ECM
NICD
SNAI2
miR34
ZEB1
CTNNB1
TWIST1
DNAdamage
p53
SNAI1
TGFbeta
p73
miR200
ZEB2
p63
ERK
CDH1
VIM
Apoptosis
AKT1
AKT2
Invasion
CellCycleArrest
miR203
p21
SMAD
CDH2
Migration
GF
EMT
Metastasis

N.

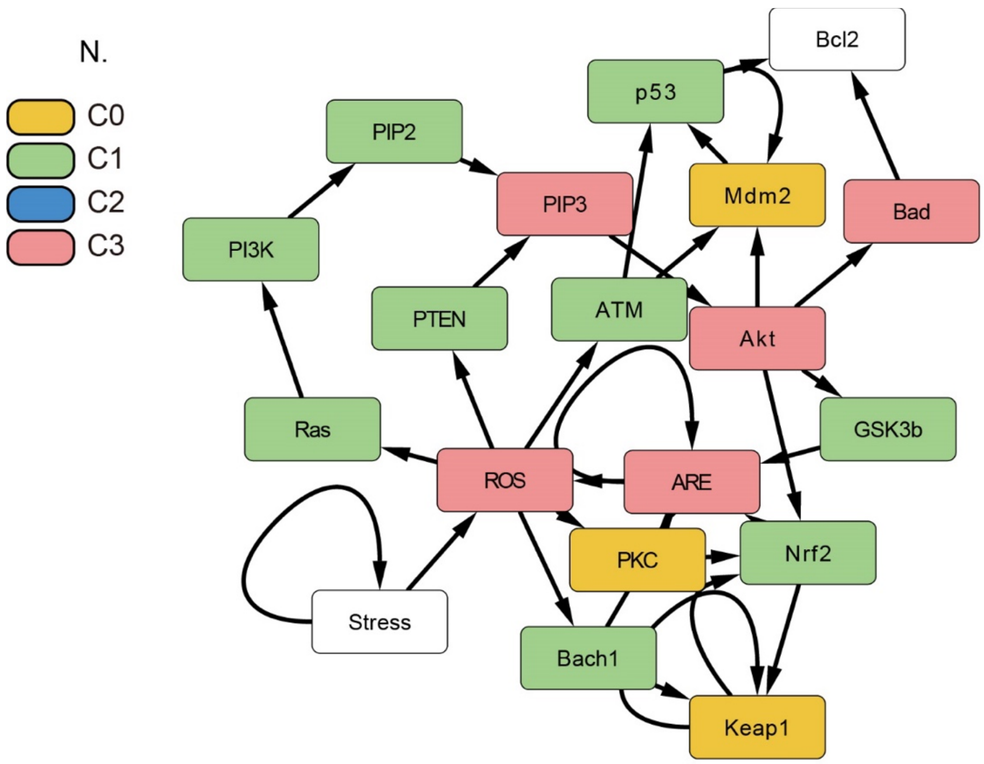
C0
C1
C2
C3
Bcl2
p53
PIP2
Mdm2
PIP3
Bad
PI3K
PTEN
ATM
Akt
GSK3b
Ras
ROS
ARE
PKC
Nrf2
Stress
Bach1
Keap1

O.

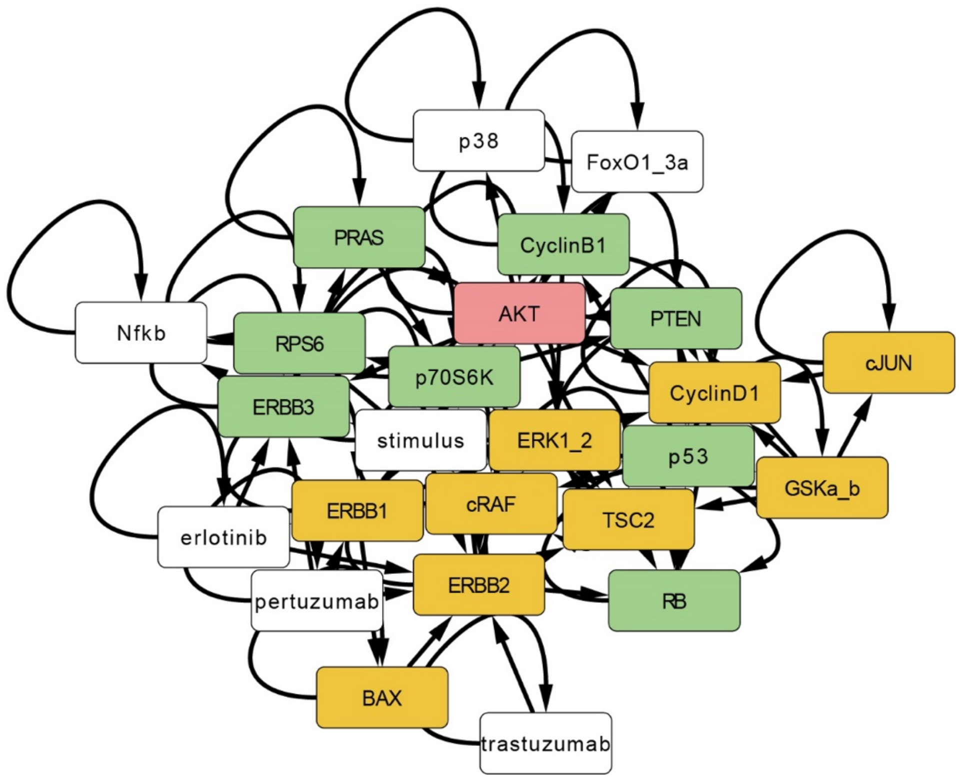
p38
FoxO1_3a
PRAS
CyclinB1
AKT
PTEN
Nfkb
RPS6
cJUN
p70S6K
CyclinD1
ERBB3
stimulus
ERK1_2
p53
GSKa_b
ERBB1
cRAF
TSC2
erlotinib
ERBB2
RB
pertuzumab
BAX
trastuzumab

P.

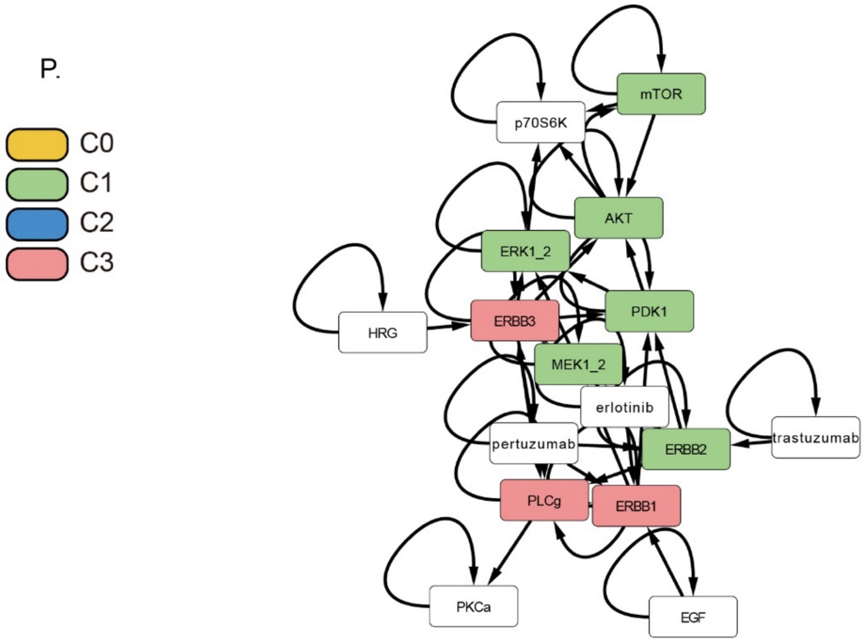
C0
C1
C2
C3
mTOR
p70S6K
AKT
ERK1_2
HRG
ERBB3
PDK1
MEK1_2
erlotinib
trastuzumab
pertuzumab
ERBB2
PLCg
ERBB1
PKCa
EGF

Q.

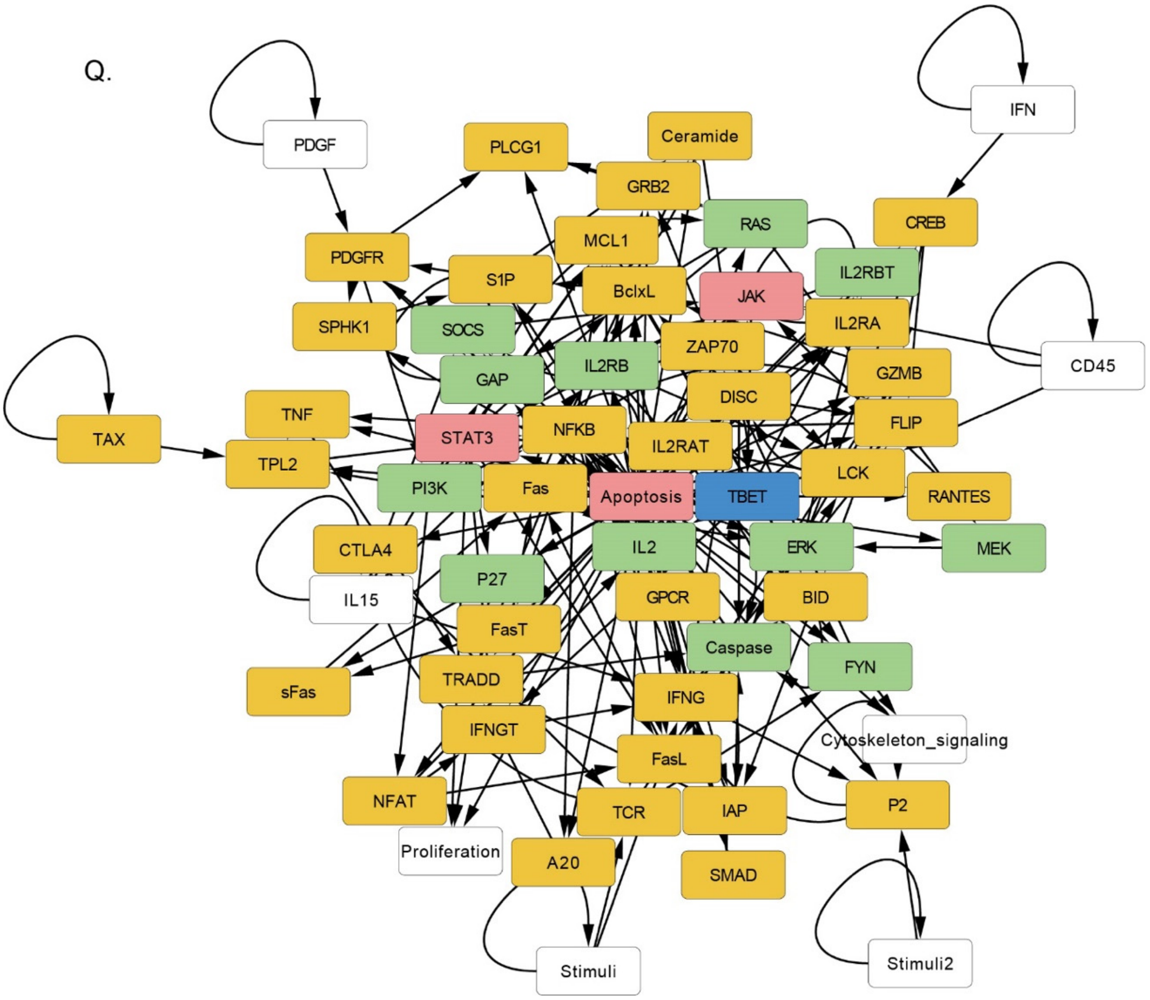
PDGF
IFN
Ceramide
PLCG1
GRB2
RAS
CREB
MCL1
PDGFR
S1P
BclxL
JAK
IL2RBT
SPHK1
SOCS
IL2RA
CD45
ZAP70
GAP
IL2RB
GZMB
TAX
TNF
DISC
FLIP
STAT3
NFKB
IL2RAT
TPL2
LCK
PI3K
Fas
Apoptosis
TBET
RANTES
CTLA4
IL2
ERK
MEK
P27
IL15
GPCR
BID
FasT
Caspase
sFas
TRADD
FYN
IFNG
IFNGT
Cytoskeleton_signaling
FasL
NFAT
TCR
IAP
P2
Proliferation
A20
SMAD
Stimuli
Stimuli2

R.

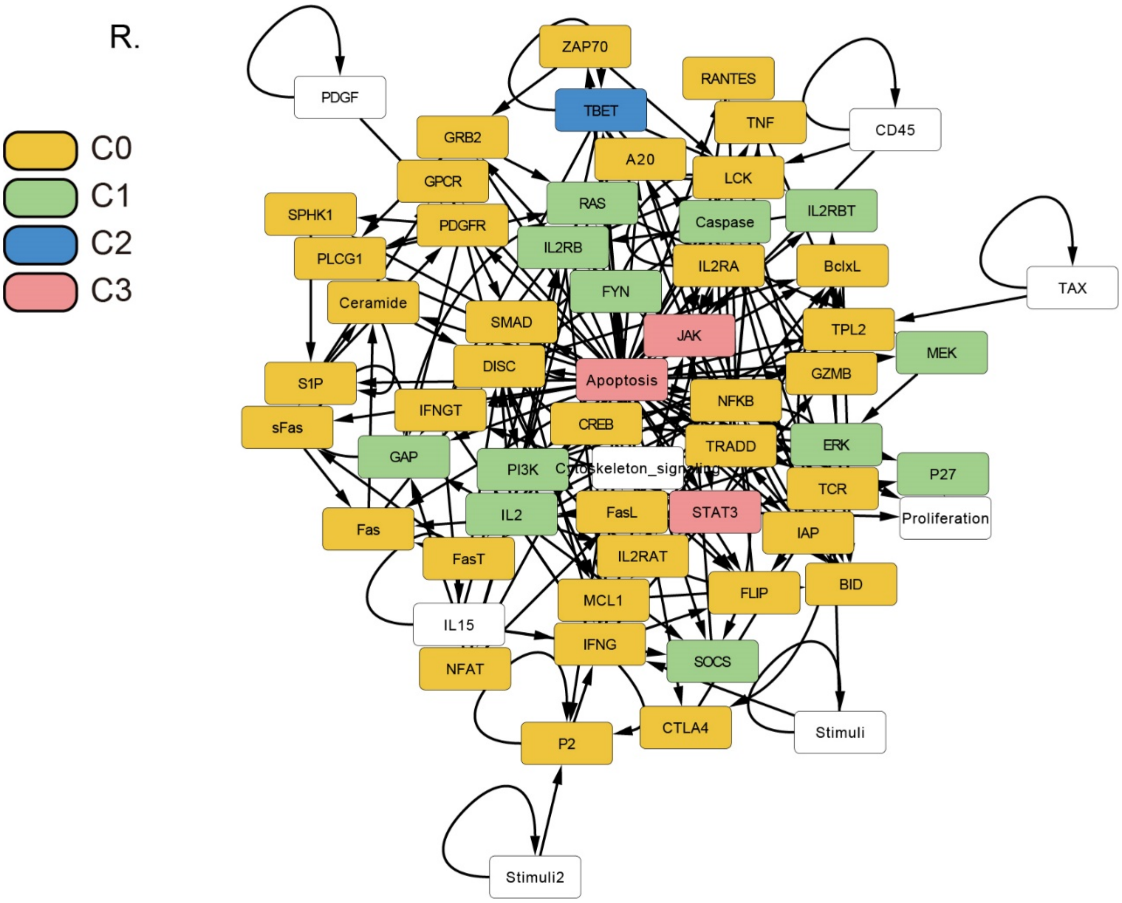

Supplementary Figure S1. **Reversibility of Cell Collective networks.** Node clasficiation of (A) apoptosis, (B) bortezomib, (C) bt474_long, (D) bt474_short, (E) colitis, (F) death, (G) fibroblasts, (H) hcc1954_long, (I) hcc1954_short, (J) hgf, (K) mammalian, (L) mapk, (M) migration, (N) oxidative, (O) skbr3_long, (P) skbr_short, (Q) tlgl_2008, (R) tlgl_2011 networks. The color of each node represents its class.

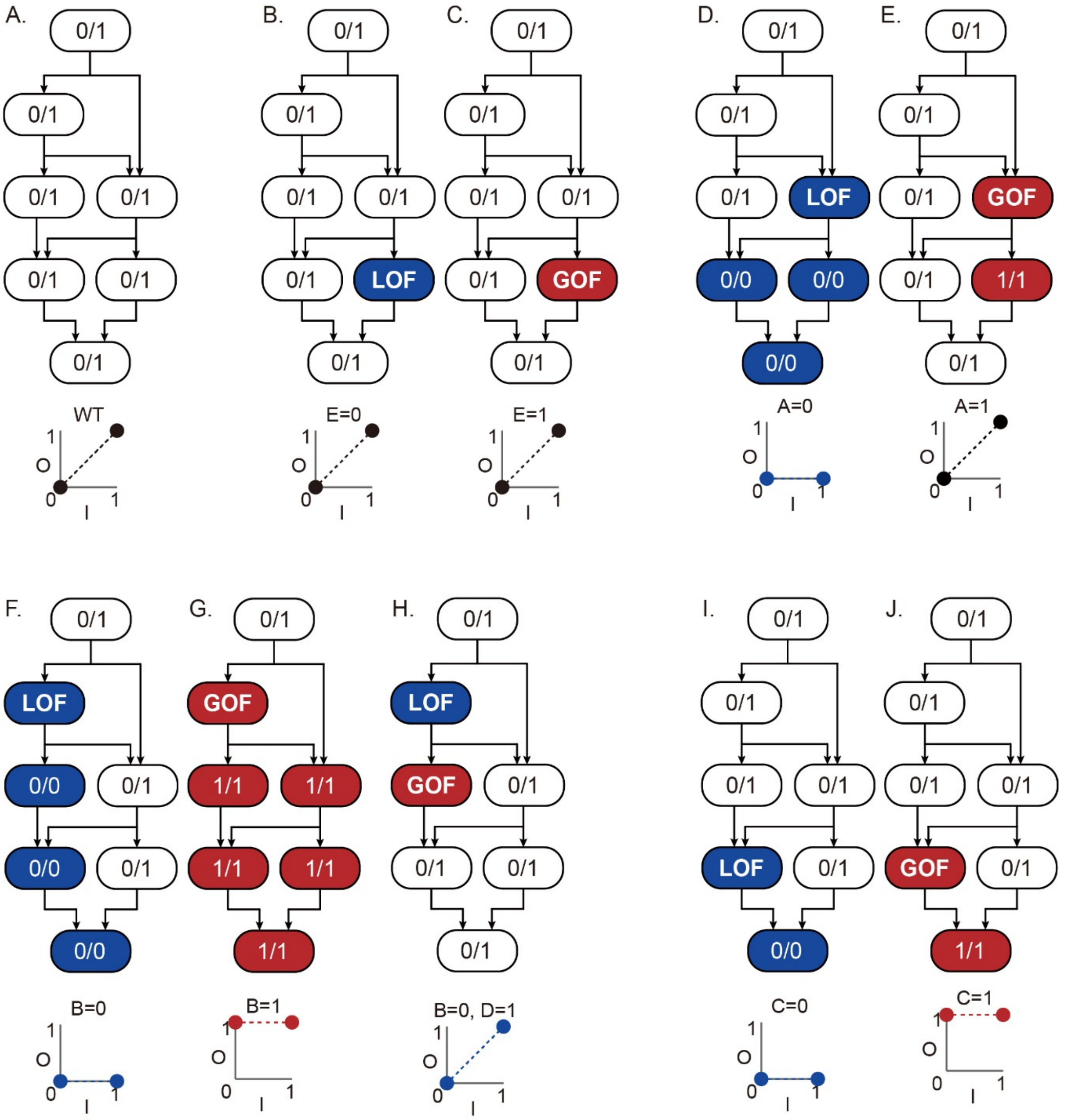

Supplementary Figure S2. **Input-output (IO) relationship of a toy network model with Boolean states**. Each node can have either "0" or "1" according to the state of an input node being turned "OFF" or "ON", respectively. (A) IO relationship of the example Boolean network, (B-J) IO relationship of the example network with various mutation conditions. LOF and GOF nodes represent the nodes with a loss-of-function and a gain-of-function mutation, respectively. Blue or red nodes represent the nodes that are determined to be 0 or 1, respectively, independent of the state of the input node.

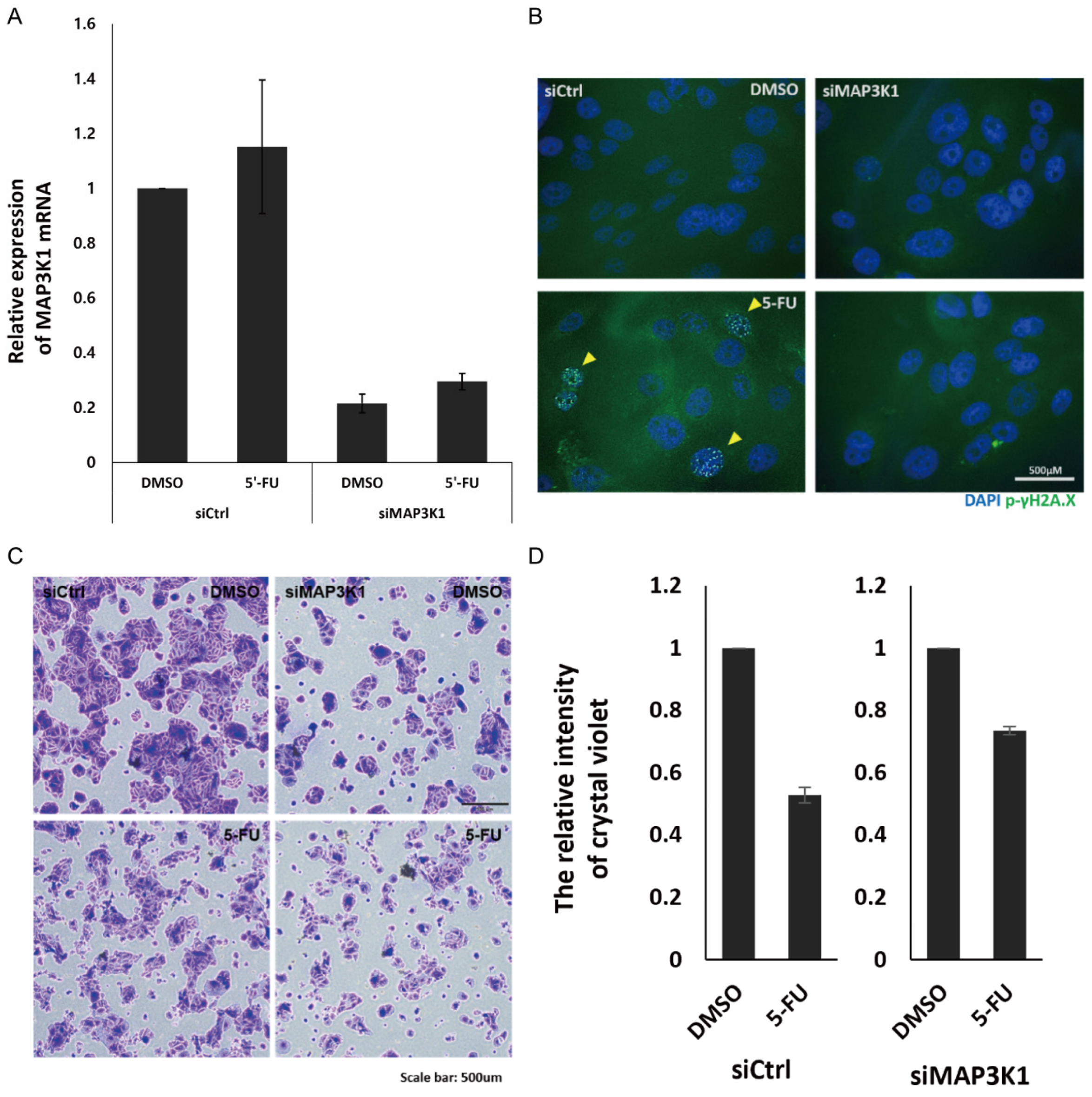

Supplementary Figure S3. Reversed DNA damage response of HT-1197 cells. (A) HT-1197 cells were transfected with siRNA against MAP3K1. (B) Transfected HT-1197 cells with control siRNAs or siRNAs against MAP3K1 were treated with 5-FU for 24hours. Then, nuclear foci were stained with antibodies which recognize p-γH2A.X and DAPI was used to stain nuclei. Green: p-γH2A.X, Blue: DAPI. (C) and (D) After grown for 24hour with DMOS or 5-FU, HT-1197 cells attached to culture plates were stained with crystal violet and solubilized crystal violet were measured

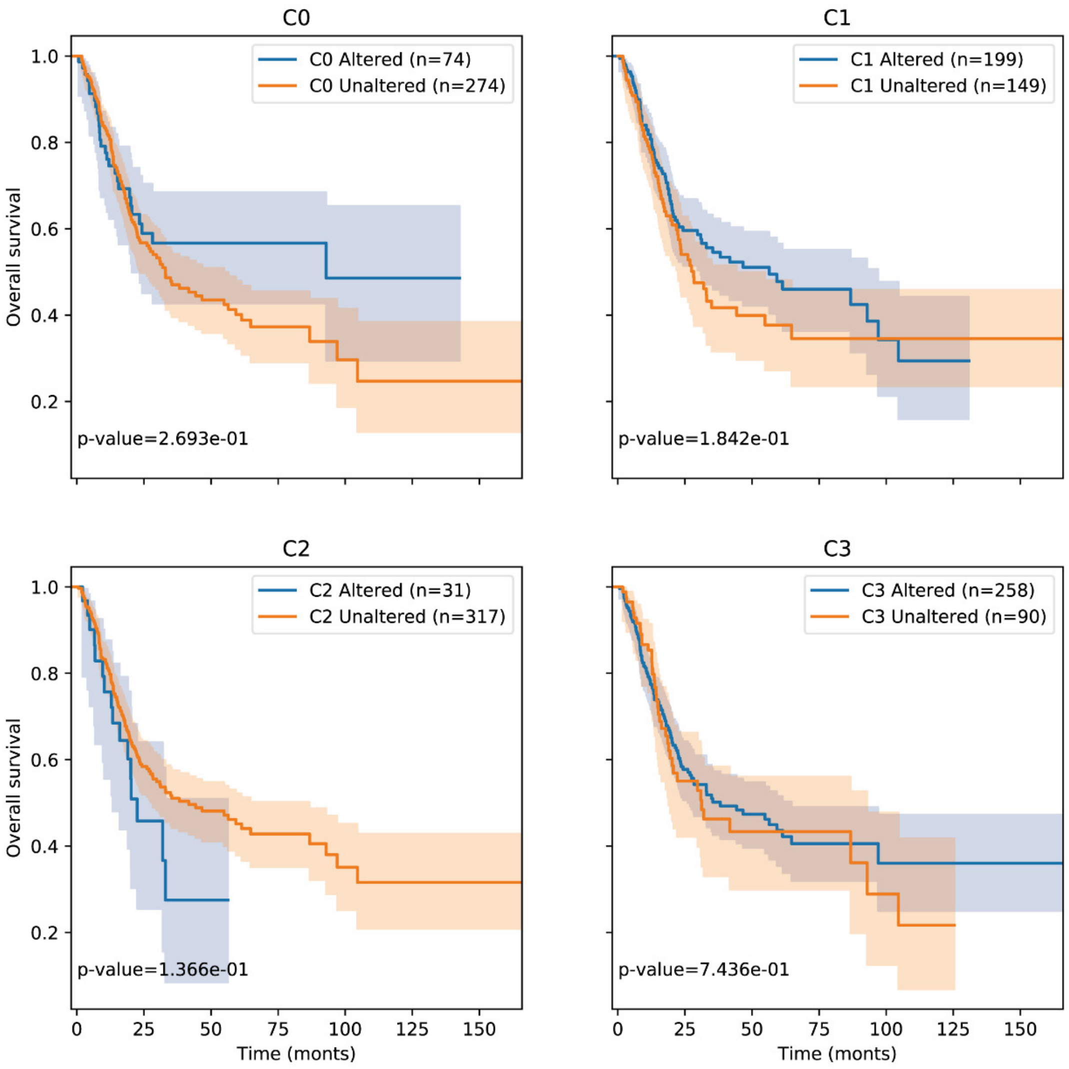

Supplementary Figure S4. Overall survival analysis of bladder cancer patients. Survival analysis of TCGA bladder cancer patients (n=348) according to alterations in each node class.

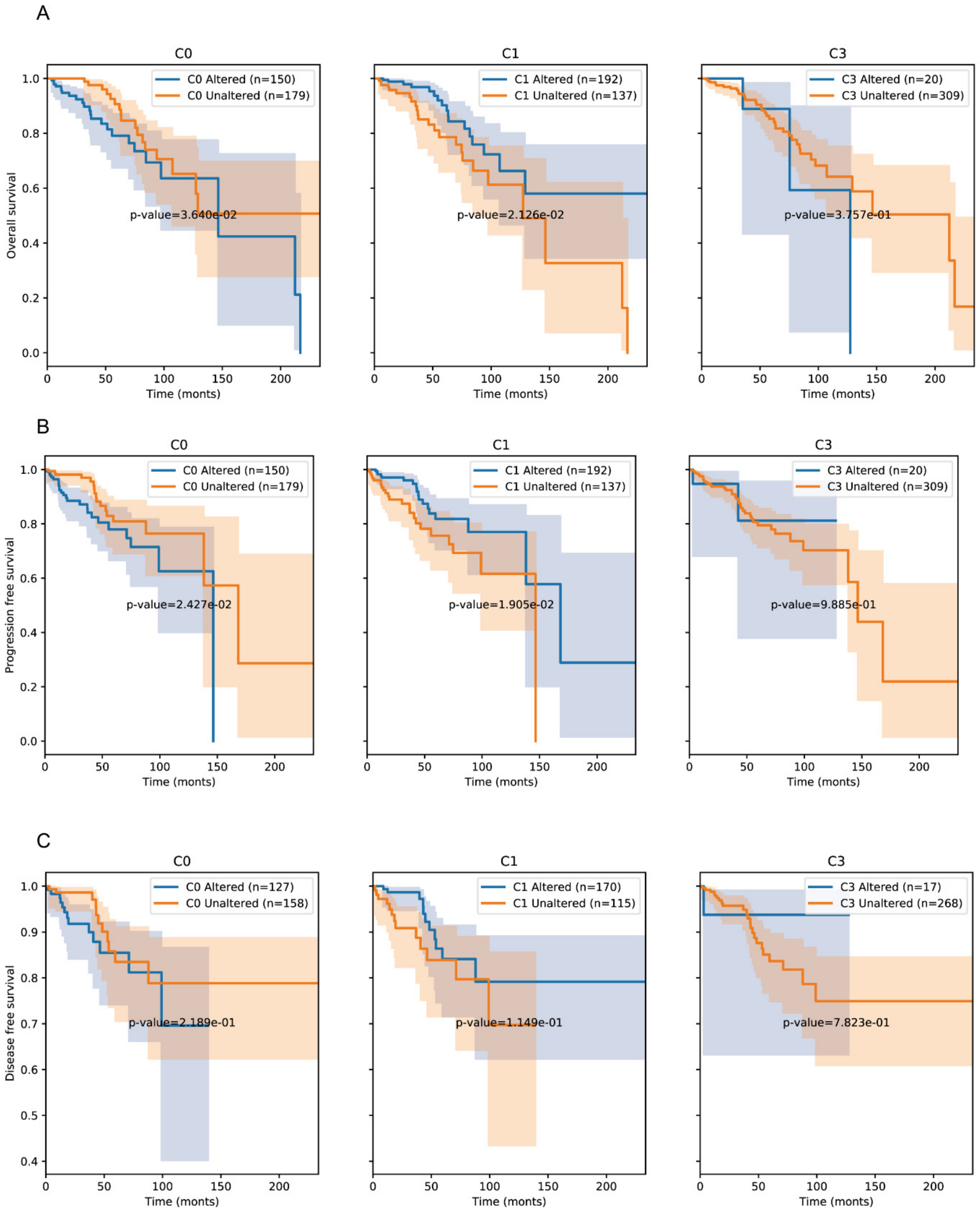

Supplementary Figure S5. Survival analysis of breast cancer patients. (A) Overall survival (n=329), (B) progression-free survival (n=329), and (C) disease-free survival (n=285) of TCGA breast cancer patients according to alterations in each node class. The breast cancer network ('mammalian' network) from Cell Collective network was utilized.

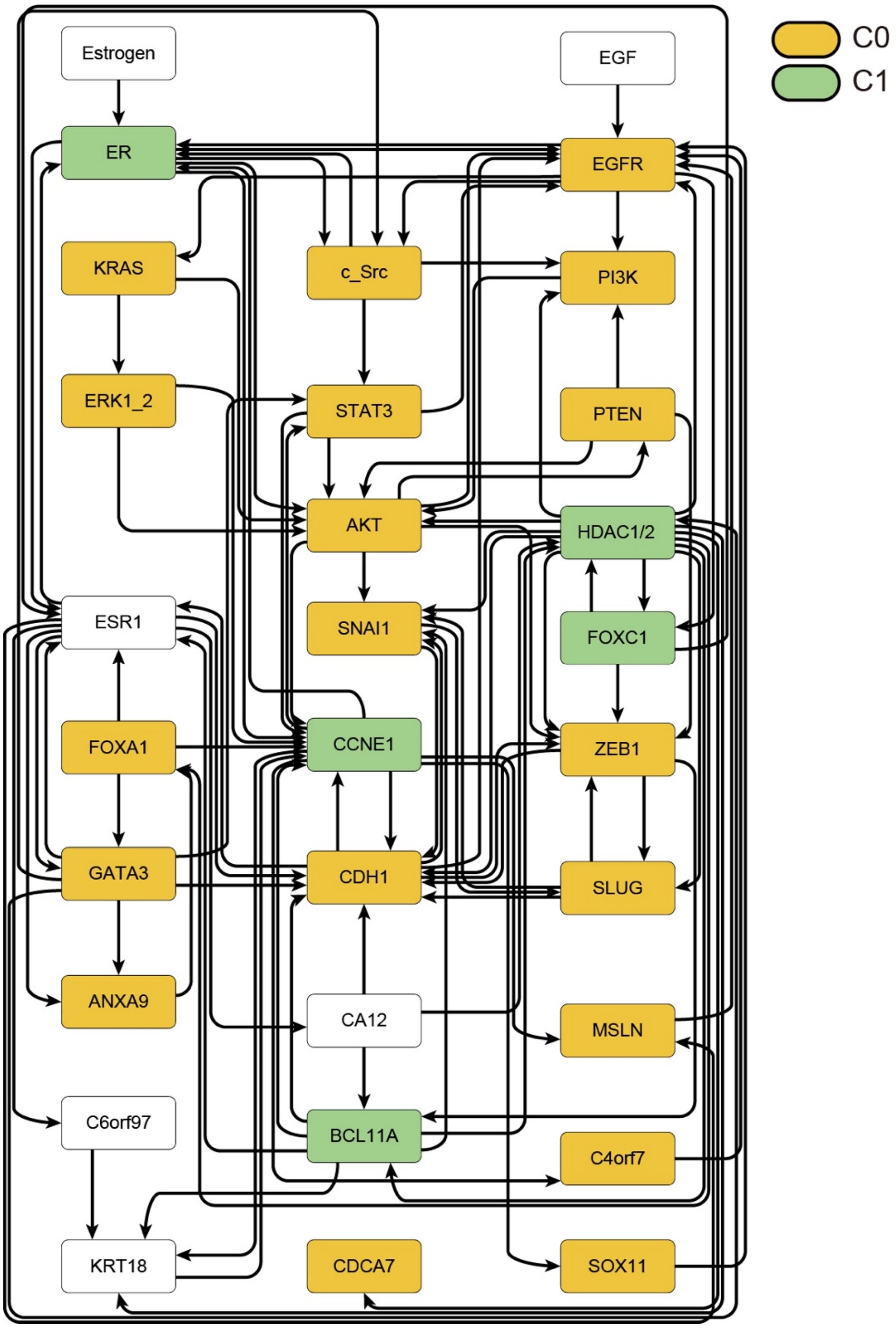

Supplementary Figure S6. Node classification of the Boolean network from Choi et al [8]. White nodes are input and output nodes; yellow nodes are C0 nodes; green nodes are C1 nodes.

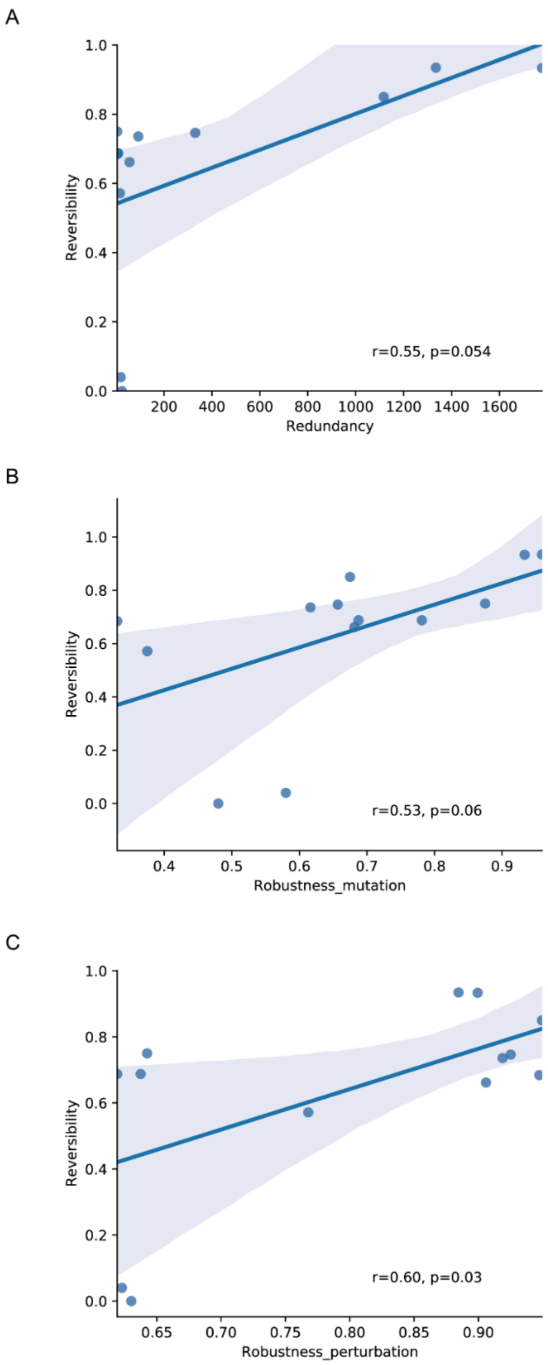

Supplementary Figure S7. Reversibility, redundancy, and robustness analysis of Cell Collective networks. (A) Reversibility and redundancy. (B) Reversibility and robustness to a permanent mutation. (C) Reversibility and robustness to a transient perturbation. Thirteen Cell Collective Boolean networks which have at least one deterministic IO matching were analyzed. Pearson correlation r and p-value are represented on each graph. The shaded area represents the 95% confidence interval for linear regression.

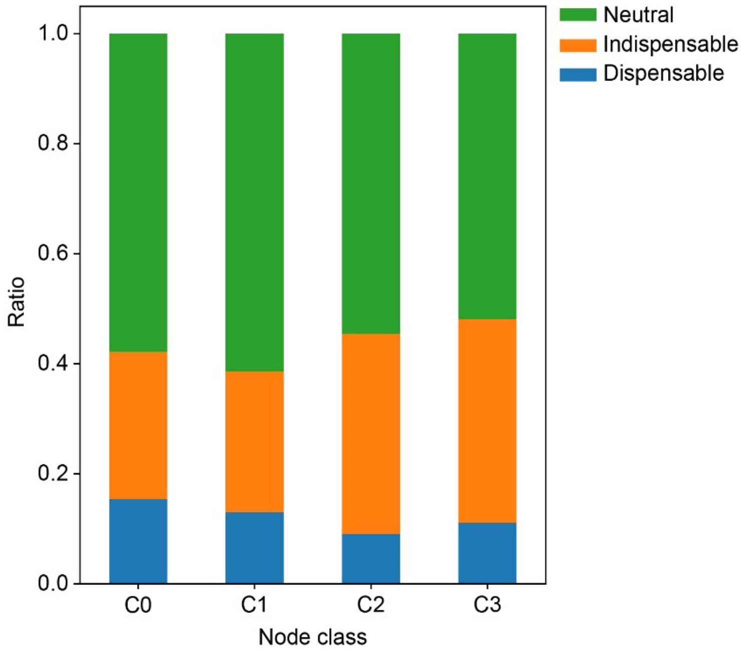

Supplementary Figure S8. **Comparison of our method and the most relevant state-of-the-art network control method.** The method of Vinayagam *et al.* [46] and that of our study are compared in classifying the nodes of 18 networks from the Cell Collective [17-30]. Each bar graph shows the ratio of dispensable, indispensable, and neutral nodes in each of the node classes obtained from our study.

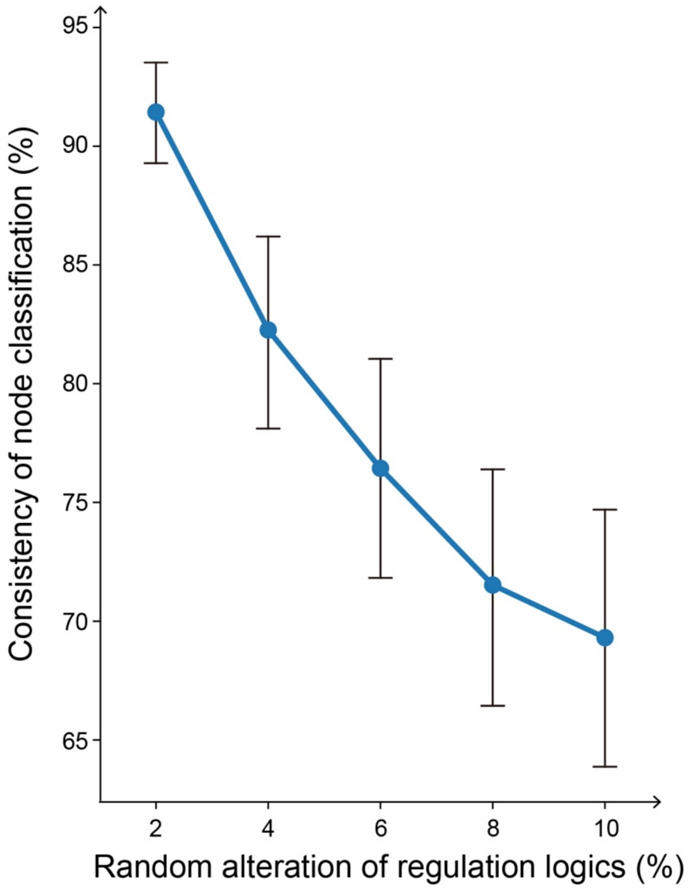

Supplementary Figure S9. Consistency of node classification under model uncertainty. Node classification shows consistent results within a small range (<5%) of random alterations in the bladder cancer Boolean network model [22]. Error bar represents standard deviation (n=100).

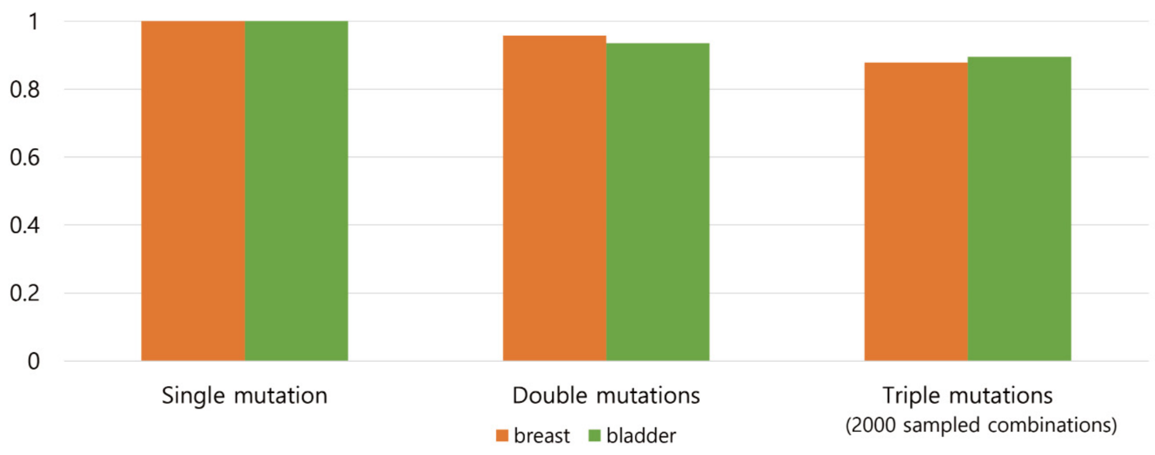

Supplementary Figure S10. Combinatorial effects of ineffective mutations on the breast ('mammalian') and bladder ('mapk') cancer networks [24, 25]. Values of y-axis represent the ratio of ineffective and reversible mutation sets in single mutation, double combinatorial mutations, and triple combinatorial mutations.

Supplementary Table S1. (separate file)

Specification of the analyzed networks from Cell Collective. *Redundancy is -1 when the network has no deterministic IO matching. Yellow rows are the selected 10 networks to generate random configuration networks

Supplementary Table S2. (separate file)

Specification of random networks. *The number of links of a random network can be different from that of the original Cell Collective network since duplicated links of random networks were removed and some other links were also removed to assure at least one input and one output node.
